# Stem Cells in Cardiovascular Medicine: Historical Overview and Future Prospects

**DOI:** 10.3390/cells8121530

**Published:** 2019-11-27

**Authors:** Mostafa Samak, Rabea Hinkel

**Affiliations:** 1 Department of Laboratory Animal Science, Leibnitz-Institut für Primatenforschung, Deutsches Primatenzentrum GmbH, Kellnerweg 4, 37077 Göttingen, Germany; MSamak@dpz.eu; 2 DZHK (German Centre for Cardiovascular Research), Partner Site Göttingen, 37075 Göttingen, Germany

**Keywords:** iPSC, PSC, ESC, cardiovascular disease, regeneration

## Abstract

Cardiovascular diseases remain the leading cause of death in the developed world, accounting for more than 30% of all deaths. In a large proportion of these patients, acute myocardial infarction is usually the first manifestation, which might further progress to heart failure. In addition, the human heart displays a low regenerative capacity, leading to a loss of cardiomyocytes and persistent tissue scaring, which entails a morbid pathologic sequela. Novel therapeutic approaches are urgently needed. Stem cells, such as induced pluripotent stem cells or embryonic stem cells, exhibit great potential for cell-replacement therapy and an excellent tool for disease modeling, as well as pharmaceutical screening of novel drugs and their cardiac side effects. This review article covers not only the origin of stem cells but tries to summarize their translational potential, as well as potential risks and clinical translation.

## 1. Introduction

Cardiovascular diseases (CVDs) remain a plight to modern-day humans, accounting for over one-third of all deaths worldwide, according to recent World Health Organization (WHO) estimates [1]. In the US alone, one person dies of CVD-related complications every 40 s, mostly ischemic attacks [2]. To this day, catheter-based or surgical interventions, e.g., coronary bypass and implantation of assist devices, are by far the most widely applied clinical measures—albeit with several complications [3,4]. Despite great improvements, most surgical interventions available are mere preservatives, i.e., attempts to sustain the functionally intact heart tissue for as long as possible without structural compensation. Howbeit, due to the progressive nature of CVDs, heart failure (HF) is, in most cases, inevitable [5]. Regardless of etiology and severity, many end-stage HF patients will eventually need cardiac transplantation [6]. With very few treatment options, not to mention the paucity of available donor hearts, the need for alternative therapeutic measures is indispensable.

In recent decades, stem cell (SC) technologies have emerged with a great promise that could be envisaged for almost all human ailments, most importantly for noncommunicable diseases characterized by organ dysfunction and/or degeneration. In this regard, CVDs are certainly the most attractive target for SC-based therapeutic approaches [7,8,9,10]. From a mere improvement of cardiac microenvironment, to partial regeneration and/or compensation of lost functional tissue, and ending with a complete fabrication of a surrogate heart, SCs have set the hopes high. Moreover, SC-based technologies have enabled great in-depth understanding of the pathogenesis of CVD entities and served as a platform to test novel therapeutic approaches at minimal risk of adverse events to patients and much lower costs. This article aims at reviewing the available knowledge on SCs and their applications for cardiovascular research, highlighting milestone achievements in both basic and translational research, and expanding in particular on pluripotent SCs. 

## 2. Adult Stem Cells

The body’s regenerative capacity is a well-ingrained piece of knowledge from ancient times. Modern science attributes this phenomenon to the presence of resident SC niches in different organs and tissue, i.e., adult SCs. These cells are undifferentiated, but they are capable of self-renewal and differentiation to one or more cell type, which sets them apart on a potency spectrum, e.g., multipotent SCs. Adult SCs’ regenerative potential becomes even more conspicuous in organs and/or tissues with high turnover rates, but, more importantly, as a response to tissue injury. A wealth of knowledge is now available on different adult SC populations, and efforts have been made to reap the benefits of these cells to treat CVDs. We highlight below a few examples of adult SCs, which declared themselves as powerful research targets for cardiovascular medicine and made their way to the clinic.

### 2.1. Skeletal Myoblasts 

Intuitively, due to embryonic and morphologic commonalities between skeletal and cardiac muscle tissues, skeletal myoblasts have been among the early attractive research targets for cardiac regeneration. Skeletal myoblasts (SM) constitute a group of satellite cell-derivatives residing within skeletal muscle fibers, which are activated upon injurious insults to migrate, proliferate, and differentiate, forming new muscle fibers, i.e., myogenesis [11]. Facilitated by their being readily accessible from autologous muscle biopsies, rapid in vitro expansion, ischemic tolerability, and low risk of tumorigenicity, the cardiac regenerative potential of SMs has been the subject of several preclinical investigations in both small and large animal models of CVDs [12,13,14,15,16,17]. Indeed, results from these studies have demonstrated positive outcomes by reducing infarct size, as well as myocardial fibrosis, thwarting ventricular remodeling and improving overall cardiac function. Consequently, several clinical trials were initiated to verify their efficacy [18,19,20,21,22]. Despite initially reported improvements in cardiac parameters of patients transplanted with SMs, many have experienced ventricular arrhythmias, which were later attributed to the lack of electromechanical coupling between the transplanted SM-derived myotubules and resident cardiomyocytes where they failed to form gap junctions [23,24,25,26]. Furthermore, larger randomized, placebo-controlled, double-blinded clinical studies not only failed to show any therapeutic benefits of SMs in patients with severe ischemic heart disease at both short- and/or long-term follow-up, but also reported postoperative arrhythmic events even upon prophylactic pharmacological treatment [27,28,29,30,31]. As a result, SMs have lost their popularity as SCs for cardiac applications. 

### 2.2. Bone-Marrow-Derived SCs

Since the mid-20th century, the BM has long been praised for its SC abundance. BM transplantation has been a clinical practice since the mid to late 1960s, intended for correction hematologic, as well as immune disorders. However, reports from the late 1990s first demonstrating the ability of BM-derived cells to migrate to injured tissues and support regeneration have instigated a wave of research on their therapeutic potentials for CVDs [32,33]. Indeed, early studies in animal models of MI corroborated the aforementioned expectations. The first tentative clinical translation of this finding was reported in 2001 in Düsseldorf, Germany, where a MI patient received autologous BM-derived nucleated cells upon catheter angioplasty and reported positive outcomes [34]. This was followed by several controlled clinical studies, albeit with inconsistent findings [35,36]. 

Generally speaking, BM-derived SCs can be sub-grouped into two large cell populations; hematopoietic (HSCs) and nonhematopoietic SCs. HSCs give rise to all blood-cell types and include a subpopulation of pro-vasculogenic endothelial progenitor cells (EPCs), which can be found in the circulating blood among others [37]. Of the nonhematopoietic BM-derived SCs, mesenchymal stromal/stem cells (MSCs) are the most studied, due to their greater multipotency, manifested in their ability to differentiate into osteoblasts, adipocyte, and chondrocytes under defined in vitro conditions, adding to their reported immune-modulatory and anti-inflammatory properties [38]. With better characterization of these cells based on surface-marker expression, studies were led, examining the therapeutic potential of each BM-derived SC type.

For example, BM-derived CD133- and/or CD34-positive HSCs were utilized for phase I and II clinical trials, where patients of MI received intramyocardial transplantation or intracoronary injections of these cells. Despite short-term follow-ups showing positive outcomes, characterized by enhanced left-ventricular ejection fraction (LVEF) along enhanced myocardial perfusion, these studies failed to show any long-term benefits [39,40]. Most recently, results from randomized, placebo-controlled, double-blinded phase III clinical trials also showed a congruent trajectory [41].

On the other hand, MSCs (CD73-, CD105-, and CD90-positive) have been a subject of greater scrutiny in both basic and translational research. Adding to their paracrine- and exosome-mediated immunosuppressive properties, MSCs are unique in their ability to evade the immune system [42]. This is largely due to their moderate levels of HLA class I expression, while lacking the expression of HLA class II, B7, and CD40 ligand conferring privilege to the immune system of their host, thus enabling allogenic transplantation without the need of concomitant immunosuppression [42,43]. Indeed, studies in large animals have shown improvements in LVEF upon MSC therapy in the setting of myocardial ischemia. Nevertheless, results from translational attempts of these findings in clinical studies fall into a wide spectrum of significance with regard to their benefits, notwithstanding their mode of transplantation (i.e., autologous vs. allogenic) [44]. Despite some showing significant improvements in patients with acute MI, other randomized controlled studies concluded no significant differences [45,46,47,48]. Nonetheless, two randomized pilot studies were conducted in 2012 and 2017 in patients with ischemic cardiomyopathy (ICM) and nonischemic dilated cardiomyopathy (NIDCM), respectively, comparing autologous to allogenic MSC therapy [49,50]. Results from these studies, also known as POSEIDON, alluded to the efficacy of MSC therapy in these patient cohorts, with superiority given to allogenic transplantation. However, these studies were limited to the small sample size and lack of a placebo control group.

### 2.3. Cardiac Progenitor Cells and Stem Cell Niches

Indeed, the heart’s endogenous regenerative capacity has been an area of extensive research over the past decades. Contrary to the long-held dogma of being a postmitotic organ, studies have challenged this notion, claiming that the mammalian heart is indeed capable of self-regeneration, albeit exiguously. Studies using mitotic index, as well as DNA labeling, have conveyed the finding that cardiomyocytes can self-renew during adulthood. However, debates have flared as to what extent this self-renewal takes place, and even to the reliability of the methods used to quantify it. Herein, nuclear labeling is not reliable, due to the characteristic polyploidy that human CMs undergo during growth or disease [51,52,53,54]. Radiocarbon (^14^C) dating, on the other hand, has provided more accurate estimates of cardiomyocyte turnover in the adult heart [55]. Interestingly, studies have shown a significant increase in cardiomyocyte count and/or ploidy in neonatal and preadolescent life in both rodents and humans, which contributed to heart growth [56,57].

Furthermore, the existence of SC niches harboring cardiac progenitor cells (CPCs) has also been reported and highlighted by research as evidence of the heart’s regenerative capacity, notwithstanding another yet-unresolved debate [51,58]. CPCs are multipotent as was shown by their ability to differentiate to cardiac cell lineages, including cardiomyocytes; they were claimed to confer cardiac tissue repair and regeneration. As a heterogeneous population of cells, they are each identified by expression of distinct markers. Of these cells, c-kit-, Isl1-, or epicardial Tbx18-positive (also WT1-positive) cells are three heavily studied cell populations due to their cardiomyogenic differentiation potential attributed during development, neonatal life, and even in adult hearts. 

The c-kit-expressing cells are the most studied CPCs, however, with contradicting reports regarding their significance for cardiac cell repair the in adult postinjury [59,60,61]. Despite their demonstrated contribution to cardiac regeneration in the neonatal hearts, c-kit-positive CPCs’ role in the adult setting of myocardial injury is largely debated [60,62,63]. A recent report alluded to the role of c-kit-positive cells in cardiac adaptation to injury, where c-kit was shown to be upregulated in response to pathological stress [64]. Furthermore, a RNA-sequencing study recently showed that c-kit-positive cells transiently adopt a cardiomyocyte-like pattern of gene expression upon myocardial infarction in vivo [65]. Contrary to these findings, more recent studies by Li and colleagues refuted the myogenic potential of these cells in the adult by using a new genetic-lineage tracing system [66]. Furthermore, the same group has shown that early segregation of myocytes and nonmyocytes during embryonic development (E10.5 to E11.5) is the cut-off line beyond which no contribution to new cardiomyocyte formation occurs, even during neonatal life [67]. Moreover, a study published earlier this year by Elhelaly and colleagues argued that c-kit-positive cells do not contribute to cardiomyogenesis, even during neonatal life [68]. Howbeit, the commonly agreed-upon consensus in the field is that CPCs are remnant SCs from developmental stages whose role in the adult heart, if any, confines to maintaining cardiac tissue homeostasis, and their cardiomyogenic potential in the context of injury is inexistent [69,70]. Importantly, however, the repercussions of the aforementioned findings instigated a wave of research endeavors to exploit the heart’s endogenous regenerative capacity for novel therapeutic interventions. In summary, the field of cardiac progenitor cells is controversy discussed, and the regenerative potential (and existence) of the cells in the adult human heart need further investigations.

## 3. Pluripotent Stem Cells

Despite the efforts that have been made with adult SCs, none of these cells could meet the expectations as a reliable treatment for CVDs. That is because not even the most potent adult SC could provide an appreciable source for myocardial tissue regeneration and/or functional compensation for the lost contractile element of the heart, e.g., as a result of infarction, let alone cardiomyopathies or congenital heart disease [71]. In this regard, the pursuit after functional CMs calls for a different type of SCs, i.e., pluripotent SCs (PSCs). 

### 3.1. Embryonic Stem Cells

**Figure 1:**
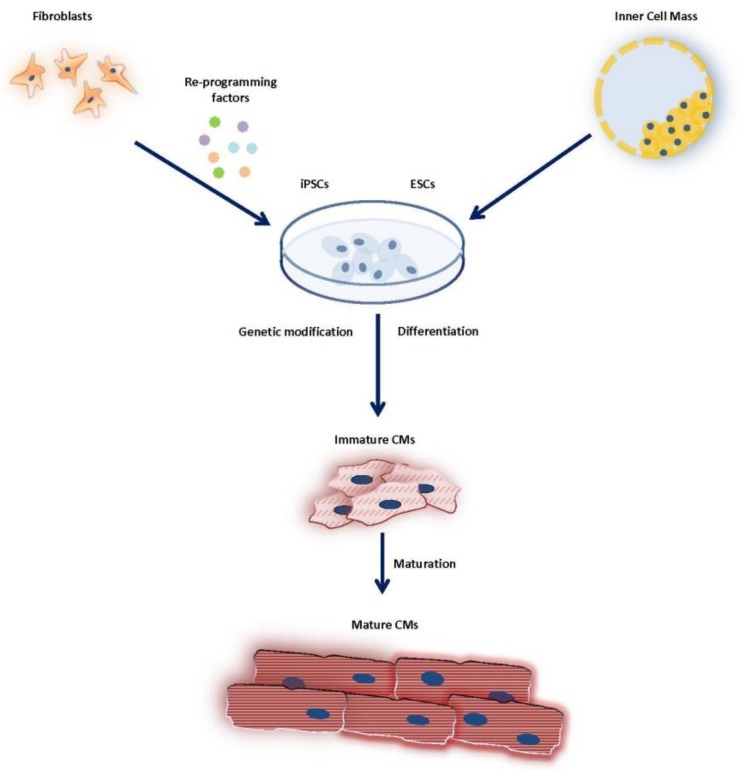
Differentiation of cardiomyocytes derived from pluripotent stem cells.

The ability of a cell to give rise of all three germ layers of the developing embryo, i.e., pluripotency, is the most vivid and sought-after character of SCs, not only in the context of regenerative medicine, but also for basic research purposes. Pluripotency of embryonic blastocyst inner mass cells was first shown in the mouse as early as 1981 by Evans et al. [72]. In 1998, Thomson et al. first reported the generation of pluripotent embryonic stem cells (ESCs, Figure 1) from human blastocysts, which are capable of self-renewal and differentiation to all three germ layers [73]. Nevertheless, ethical considerations have long hovered over human ESCs (hESCs), as their derivation entails destruction of an embryo. This has prompted legislative issues, in that many countries have imposed bans on their use and/or research funding [74,75]. To add insult to injury, ESCs’ ability to form teratomas (tumors of mixed germ layers) when transplanted undifferentiated has further flared the argument against their clinical application, despite efforts to enhance differentiations and purifications protocols [76].

### 3.2. Induced Pluripotent Stem Cells

It was not long until the not-so-bright picture of SC research changed. Inspired by the preexisting knowledge of master regulator genes capable of imparting cellular identities, Takahashi and Yamanaka developed the first technique of somatic cell reprogramming in 2006 [77,78]. In their Nobel Prize experiment, induced pluripotent stem cells (iPSCs, Figure 1) could be generated from somatic cells, such as skin fibroblasts, by expression of four transcription factors that were found to be crucial for cellular reprograming to ESC-like inner mass cells, namely Oct3/4, Sox2, c-Myc, and Klf4 [78]. Ever since, scientist have raced to improve the reprogramming efficiencies of iPSCs by manipulating the transcription-factor cocktail and selecting for expression of other transcription factors, such as Nanog and Lin28 [79,80,81]. Generation of viable and tumor-free whole organisms with iPSCs that were capable of germ-line transmission was also made possible [82]. Unsurprisingly, human iPSCs (hiPSCs) were generated as soon as one year after their first generation in a mouse, and by the same pioneering group of scientists, as well as others [83,84]. 

### 3.3. Embryonic Stem Cells Versus Induced Pluripotent Stem Cells

The primary intended purpose of reprogramming of somatic cells and generation of iPSCs was to wipe the initial cellular identity and drive them back to the embryonic inner mass state, and hence serve as a surrogate for embryonically derived cells, i.e., ESCs. Indeed, iPSCs greatly resemble conventional ESCs in terms of growth characteristics, gene-expression profiles, epigenetic status, and developmental potential, which were shown in earlier studies by Yamanaka and colleagues, as well as others [79,84,85,86]. However, upon comparison of various undifferentiated cell lines, reports argued that iPSCs may not be perfectly identical to conventional ESCs. This is largely attributed to the unique epigenetic signatures of their parent somatic cells. Despite previous studies showing that somatic cells undergo epigenetic remodeling upon reprogramming, studies have shown that iPSCs indeed retain epigenetic patterns of their donor cells, e.g., CpG island methylation [86,87,88,89,90]. Furthermore, gene and miRNA expression signature were also shown to trail along with iPSCs (reviewed in [91]). Upon differentiation to CMs, further comparison of mature CMs differentiated from ESCs and iPSCs can be insightful. In this regard, CMs of either origin were reported to display similar ultrastructural phenotypes, upon electron microscopic examination [92]. In line with these findings, a study by Gupta et al. revealed that global transcriptional profiles of mature CMs derived from either human iPSCs or ESCs are highly similar [93]. However, iPSC-CMs were more likely to share some somatic cell signature with their undifferentiated iPSC-parents. Thus, identification of these variations between iPSC- and ESC-CMs, as well as the interline variability of either type of PSCs, is essential before they are utilized for disease modeling or clinical application.

Unlike ESCs, iPSCs derivation does not involve destruction of embryos, and hence does not fall into the same ethical pitfalls. However, other ethical considerations arose with hiPSCs, especially with regard to the possibility of reproductive cloning, the risk of generating genetically engineered human embryos, and, more extremely, human–animal chimeras [94]. Furthermore, and like ESCs, iPSCs are subject to safety concerns due to their ability to form tumors, even with rigorous protocols of differentiation and selection [95].

In recent years, substantial developments in stem cell technology in terms of reprogramming efficiency and enhancing their clinical applicability have prompted scientist to utilize pluripotent stem cells (PSCs), not only to regenerate, but also to model the human heart for basic research purposes. Furthermore, some countries have tentatively started to loosen their tight regulations, especially on hESCs; a step that coincided with the establishment of stem-cell registries in the US and Europe [96,97,98]. This has led to several initiatives on stem cell therapy for many disease conditions, including CVDs [99]. As promising as this may sound, several challenges, however, preclude the full realization of PSC-based therapy. In the following, we shall focus on PSCs by addressing efforts made over the past decades to optimize their generation, differentiation, and maturation for CVD research, as well as efficient delivery methods for late clinical and/or translational purposes.

## 4. Cardiac Stem-Ness

Embryology is the fundament for generation of cardiac cells from PSCs in the laboratory. The heart is the first organ to develop and function during embryogenesis [100]. In the lateral mesoderm, cardiac specification takes place, a process initiated by two T-box transcription factors, Eomesodermin and Brachyury(T), which have been shown to induce the expression of yet another critical factor, namely mesoderm posterior 1(MesP1) [101,102]. MesP1 is a basic helix-loop-helix (bHLH) transcription factor considered to be the master regulator orchestrating the differentiation and commitment of cardiac precursors [101,102]. Cardiac precursors then assume a crescent-shaped structure known as the cardiac crescent, at which cells are irreversibly committed to the cardiac lineage. This is marked by the expression of key transcription factors, namely Nkx2.5, GATA4 and Tbx5 [103]. Two waves of Nkx2.5 expression ensue, depicting the formation of two regions known as the first and second heart fields, which subsequently give rise to different heart chambers, as well as the cardiac outflow tract [76,103].

After all, heart development is a dynamic three-dimensional process governed by an intricate network of signals and gene transcription [104,105]. Howbeit, three major signaling pathways converge to drive the process, from early cardiac tissue specification of mesoderm progenitors to subsequent differentiation into cardiac progenitors, namely BMP (bone morphogenic protein) and Nodal/Activin, both being members of the TGF-β (transforming growth factor beta) cytokine family, and the Wnt/β-catenin [106,107]. Paracrine signals responsible for the fine-tuning of those pathways is crucial for heart development. For example, signals activating the Wnt/β-catenin pathways are essential for early mesoderm induction, whereas inhibitors of the same pathway are subsequently required for precardiac specification [76]. 

### 4.1. Generation/Differentiation of Pluripotent Stem Cells 

Protocols for in vitro generation of cardiac stem cells (CSCs) from PSCs, either from ESCs or iPSCs, rely primarily on simulating the signaling microenvironment, which induces the aforementioned rudimental pathways, starting by initial epithelial to mesenchymal transition, mesodermal specification, and subsequent cardiogenic differentiation, followed by selection for cardiac markers [108,109,110]. The initially reported protocols relied simply on serum in culture medium as a source of inducing factors, observing spontaneous formation of aggregates called embryoid bodies (EBs) when cells are plated in suspension [111]. These EBs would later show contractions and positive staining for cardiac markers. This method was first reported in ESCs, however, with very low efficiency [111]. Nevertheless, the EB-based differentiation remained a standard protocol and was also the first differentiation method applied to generate CMs from mouse iPSCs only a couple of years after their first introduction in 2006 [112,113]. The first CMs generated from iPSCs were reported by a team of researchers from Leibniz institute in Germany, with few refinements introduced to the protocol, which led to the differentiation of typical CMs comparable to those generated from ESCs [112]. Interestingly, precisely at the same time and in the same journal issue, the iPSCs-founding team from Kyoto also published a systematic differentiation protocol of mouse iPSCs into cardiac lineages [113]. Nevertheless, and as mentioned before, the efficiency of the EB-based protocols was low, mainly due to the uncontrolled differentiation cues in the supporting media. One of the earliest and most cited protocols to differentiate ESCs to beating CMs was reported by Mummery et al. in 2003, where they delegated the differentiation cues to paracrine signaling of murine visceral endoderm-like cells (END-2) [114]. They compared their generated CMs to primary human fetal CMs, as well as primary human adult CMs, and reported comparable structural and functional properties. Improvements to differentiation protocols by temporal application of cytokines, as well as small molecule inhibitors (e.g., inhibitors of the Wnt pathway) to simulate the developmental processes have also been successful introduced to generate CMs from PSCs [108,109,110,115,116,117]. Furthermore, several groups have sought to simplify the differentiation protocols by using chemically defined culture media consisting of only a few components [115,116,117]. 

Nevertheless, differentiation of PSCs by using standard protocols usually yields a mixed population [118]. Thus, identification of selection markers is crucial for the purification of cardiomyocyte progenitors. Pioneering studies by Moretti and colleagues have greatly contributed to the refinement of selection protocols for cardiac organogenesis from PSCs [119]. From an embryological standpoint, myocyte progenitors are distinguished from nonmyocytes (vascular progenitors) by consistent expression of Isl1-1 transcription factor, along with Nkx2.5, whereas co-expression of Isl-1 and CD31 is a marker for endothelial progenitors [119]. Among myocytes, cardiomyocytes can be further distinguished from smooth muscle cells (SMCs) by expression of vascular cell adhesion molecule 1 (VCAM 1) and signal regulator protein alpha (SIRPα), both of which were reported to be reliable selection markers in culture conditions, yielding as much as 98% pure-cardiomyocyte populations by antibody-based sorting from PSCs [120,121]. Successful differentiation can be further confirmed by expression of other cardiomyocyte markers, such as cardiac troponins, e.g., TNNI1 [121,122]. Using lentiviral vectors, expression of selection markers, e.g., antibiotic-resistant genes or fluorescent proteins, under control of cardiomyocyte-specific promoter, has also been reported to purify cardiomyocytes [123,124]. Importantly, documented biochemical disparity between CMs and non-CMs in energy metabolism was also exploited for the so-called “metabolic purification” of CMs. In this regard, manipulation of culture conditions by altering the composition of the culture medium (e.g., glucose depletion, lactate, and glutamine supplementation) was found to be crucial for such nongenetic purification of CMs [125].

Finally, studies have pointed out the important role of MicroRNAs (small, noncoding RNAs that regulate gene expression by degradation of messenger RNAs) in CM phenotype differentiation [126,127,128].

### 4.2. Maturation of Pluripotent Stem Cells 

To be utilized for disease modeling or regenerative medicine, one might expect PSC-CMs to recapitulate the structural and functional characteristics of adult CMs (Figure 1). Nevertheless, CM differentiation of PSCs usually yields immature cells, resembling the embryonic or fetal state [129]. This manifests in their morphology, gene expression, and electrophysiology. More recently, single-cell-transcriptomic analyses have proven to be a powerful tool to understand the transcriptional roadmap of in vitro CM differentiation, and therefore enable a better design of differentiation and maturation protocols [130,131]. The following highlights the major differences between immature PSC-derived CMs and mature and/or adult ones. 

Morphologically, PSC-CMs are significantly smaller in size, compared to their adult or matured counterparts. Upon maturation, cells assume an elongated shape, reminiscent of adult CMs [132]. Sarcomeres are much less organized in immature PSC-CMs and become much more organized upon maturation, which usually correlates with isoform switch of sarcomeric proteins. A good example is troponin I, wherein different isoforms distinguish embryonic CMs from adult ones [133,134]. Stoichiometric replacement of the fetal troponin *TNNI1*, encoding slow skeletal troponin I (TnIs), gene with the adult *TNNI3*, encoding adult cardiac troponin I (TnIc), gene was reported in a study by Bedada et al. as a quantifiable marker for maturation in PSC-CMs [135]. Another well-characterized hallmark of mature CMs is the isoform switch of myosin heavy chain (MHC). Two isoforms exist, the alpha isoform (encoded by *MYH6*), also known as the faster isoform, and the beta isoform (encoded by *MYH7*), also known as the slower isoform [136]. Importantly, differences exist between rodents and humans in this regard. In small rodents (mice and rats) with faster heart rates, alpha-MHC isoform predominates and increases upon maturation, whereas, in bovine and human hearts, despite the presence of the alpha-MHC isoform, the beta MHC isoform usually predominates, regardless of the state of development, and increases with age [136,137]. However, most differentiation protocols of human PSC yield CM with both isoforms, but studies have shown that long-term cultures, especially on stiff substrates, lead to a greater shift toward the beta-isoform, reflecting maturation [138]. Titin is another key component of the sarcomere that undergoes isoform switch during maturation. Fetal titin isoforms N2BA 1 and 2 are more compliant, but they switch to the N2B isoform in postnatal and adult cardiomyocytes [139]. Genes encoding structural and force-generating myofibrillar proteins are much poorly expressed in in vitro maturated PSC-CMs when compared to adult- and fetal-heart samples [140]. This might be attributed to the absence of biomechanical stresses in vitro, which are normally present upon heart development in vivo [141].

Electrophysiological, and similar to contractile components, ion-transport related genes, such as those for voltage-gated potassium channels, e.g., *KCNJ2* and Ryanodine receptor *RYR2*, were poorly expressed in immature CMs [142]. The lower expression level of the *KCNJ2*-encoding membrane protein of the inward-rectifier current, as well as genes encoding beta-subunit members of the voltage-gated potassium channels, such as *KCNIP2*, *KCNAB1,* and *KCND3*, all affect both the inward-rectifier (I_k1_) and the transient-outward (I_to_) currents, respectively, leading to the characteristic “less negative” resting membrane potential in PSC-CMs (~–60 mV) compared to adult CMs (~–90 mV) [129,132,142,143]. Furthermore, studies have shown that PSC-CMs have few to no T-tubules, which are key components of excitation–contraction coupling (ECC) and a hallmark of mature and/or adult CMs; this is typified by unsynchronized Ca^2+^ transients in immature CMs [144].

Metabolically, immature CMs have few and underdeveloped mitochondria, accounting for a small fraction of the cell volume. Adult CMs, on the other hand, show highly developed, well-distributed, and dense mitochondria, accounting for ~20–40% of the adult myocyte volume. During development, hypoxia is an early trigger for mesoderm cardiac specification [145]. The growing heart, thus, resorts to glycolysis as a major source (80%) of energy. As CMs mature and become terminally differentiated, mitochondrial oxidative capacity increases, with fatty acid β-oxidation (80%) becoming a major source of energy [146]. PSC-CMs recapitulate both mitochondrial structure and glycolytic dependence of embryonic-state CMs [92,147]. Recent studies have shown that tweaking the culture media composition to mimic these metabolic changes, e.g., replacing high-carbohydrate, high-insulin, glucose-based, with low-carb, low-insulin fatty-acid-based media-enhanced maturation [148]. Table 1 summarizes the major differences between human-PSC-derived and adult CM.

Over the years, efforts have been made to enhance the maturation of PSC-CMs, and these include prolonged cultures, using stiff gel micro-patterned substrates, and application of electrical and/or biochemical stimuli [132,149,150,151,152]. The overall goal was to simulate the in vivo environment of the myocardium, where CMs are under constant physical, topographic, and humoral stimuli leading to their structural and functional maturation. 

### 4.3. Engineered Heart Tissue

Importantly, the accumulated knowledge of cardiac stem cell biology and maturation has culminated in the so called “Engineered Heart Tissue” (EHT), a milestone achievement. The nascent EHT is attributed to work done by Zimmermann and Eschenhagen in the early 2000s [153,154]. Ever since, EHT technology has rapidly progressed through refinements in mechanical loading, electrical stimulation, medium supplementation, and miniaturization. The result was a 3D cardiac tissue structure with mature CMs and near-physiological contractile forces [155]. The pioneering work of these scientists has opened the doors for more revolutionary developments, such as 3D bioprinting, organ-on-chip platforms, and laser-cut decellularized myocardium, all with ample opportunities for both basic research and clinical applicability [156,157,158].

## 5. Applications of PSCs in Cardiovascular Research

### 5.1. Pluripotent Stem Cells in Cardiovascular Disease Modeling

The use of PSCs to model cardiac disease in vitro has become highly attractive, especially after the introduction of iPSCs [107]. This is mainly because of inadequacies of other models in terms of sampling, propagation, and maintenance, as for human primary cardiomyocytes, or their ability to fully recapitulate physiological properties of human CMs, as in rodent models. Considering the relative difficulty in cloning and genetically modifying human ESCs, most established models of CVDs are iPSC-based [107]. The feasibility in sampling and propagation of iPSCs, as well as advances in reprogramming protocols, which later adopted nonintegrating genomic approaches to deliver the reprogramming factors, has greatly increased their popularity [159]. Patient-specific iPSC-CMs have enabled the study of genetic variants underlying several CVDs and establish a phenotype–genotype understanding of not only monogenic, but rather complex and difficult-to-model genetic variants (e.g., chromosomal deletions or translocations), and, most important, model congenital heart disease (CHDs) in newborns [160,161]. As a result, several patient-derived iPSCs lines have been developed to model CVDs. The first of such was reported by Carvajal-Vergara et al. in 2010 for LEOPARD syndrome, an autosomal dominant developmental disorder characterized by hypertrophic cardiomyopathy [162]. Ever since, several other cell lines have been reported, mainly modeling cardiac channelopathies, (e.g., long QT syndromes), cardiomyopathies of wide etiology spectrum (e.g., dilated, hypertrophic, arrhythmogenic, Barth syndrome, and Pompe-disease-associated), and infectious myocarditis [163,164,165,166,167,168,169,170,171,172,173,174,175,176,177].

Despite the previously discussed disparities in structural and electrophysiological characters of iPSC-derived and adult CMs, these studies have shown that patient-specific iPSC-CMs recapitulate their corresponding disease phenotypes. For example, whole-cell patch-clamp analyses of different long QT syndrome (LQTS) patient-derived iPSC-CMs showed typically prolonged APs, decreased rectifier potassium currents I_K_, increased late sodium currents I_NaL_, and impaired voltage-dependent inactivation of the L-type channels (LTC), due to malfunctions in corresponding proteins of potassium (KCNQ1, KCNH2 in LQTS1 and 2), sodium (SCN5A in LQTS3), and calcium (CaV1.2 in LQTS8 or Timothy syndrome) channels, respectively. Moreover, these patient-specific models demonstrated great utility for pharmacological screening of several drugs with disease-modifying abilities, leading to both novel and/or personalized therapeutic strategies (reviewed in [160]). 

Finally, patient-specific iPSC-derived non-CMs were also generated, for example, of SMCs or endothelial cells. A more recent example is an elegant publication by Gu et al., utilizing iPSC-derived endothelial cells from patients with autosomal-dominant mutations in *BMPR2* associated with familial pulmonary arterial hypertension (FPAH) [178]. In their study, comparing symptomatic patients with unaffected carriers highlighted important modifiers of the BMP-receptor pathway, as well as differentially expressed genes, which imparted protection against FPAH. Their findings were of great importance as to the identification of multiple genetic factors affecting disease penetrance, which could be therapeutically targeted to modify disease progression and severity.

Importantly, the previous example behooves an important consideration when conducting studies on patient-specific iPSCs for CVD modeling, which pertains to the identification and/or the availability of proper control lines. This is because, even among patient-matched donor cohorts, genetic variability can still confound the analysis of the disease phenotype, especially in the presence of disease modifiers, or when the genotype–phenotype is less conspicuous [169,179]. In such cases, it is possible to rely on more than one control cell line—albeit a laborious approach. Alternatively, the patient’s iPSC-CMs can be compared to those from a healthy sibling, thus limiting genetic variability [171]. However, recently developed computational in silico models of iPSC-CMs and their optimization by Paci and colleagues have provided an unprecedented approach to this issue, enabling simulation and calibration of over a thousand diseased or control iPSC-CM models [180,181,182]. Finally, in case of monogenetic diseases, an isogenic cell line created by correction of the disease-causing mutation in the patient iPSCs by means of gene-editing approaches can serve as the best control cell line (discussed below). An elegant example was reported in a study by Bellin and colleagues, where they used iPSC-CMs from LQTS2 patients with a distinct mutation in potassium channel KCNH2, and compared it to an isogenic control upon correction of the genetic mutation [183]. Furthermore, they reproduced the study model in human ESC-CMs, where they introduced the same mutation, and recapitulated the disease phenotype, thus generating two genetically distinct isogenic pairs of LQTS2 and control lines.

### 5.2. Pluripotent Stem Cells in Pharmaceutical Screenings 

Since their first introduction, iPSC-CMs have become attractive for drug testing, antiquating the hERG test, which utilizes cell lines that stably express the human ether-a-go-go-related gene (hERG) *KCNH2* encoding the I_Kr_ channel involved in cardiac repolarization. Whole-cell patch-clamp screening for compounds that block the I_Kr_ current serves as a good marker of cardiotoxicity, as such blockade leads to the prolongation of the QT interval, i.e., ventricular repolarization, resulting in potentially fatal ventricular tachycardia called Torsade de Pointes [184]. Since the actual risk for cardiac toxicity is not confined to a certain channel and/or mechanism, iPSC-CMs are hence more representative in typifying cardiac toxicity to drugs. Furthermore, recent introduction of automated patch-clamp (APC) devices, all-optical cardiac electrophysiology with novel optogenetic actuation, and video microscopy have all revolutionized drug screening in iPSC-CMs and tissue constructs, enabling high-throughput testing platforms for hundreds of samples and/or drugs, thus creating a wealth of information in short time [185,186,187,188]. Furthermore, comprehensive in vitro proarrhythmic Assay (CIPA) has recently emerged as a powerful model to predict cardiac toxicity by integrating the knowledge from both in vitro and recently developed in silico computational models (http://cipaproject.org/about-cipa/) [189]. However, as discussing this is beyond the scope of this review, we refer the reader to the cited work by Paci et al.

### 5.3. Genetic Modification of Pluripotent Stem Cells

The advent of genome-editing methods has incited great progress in PSC research. Exploiting the cell’s inherent DNA-repair mechanisms, such as nonhomologous end-joining (NHEG) or homologous recombination (HR), has long been used to introduce small but disruptive mutations to target genes, either by insertion or deletions of base pairs, also known as “Indels”. The discovery and later advances of nucleases that can more specifically target desired sequences, such as zinc-finger nucleases (ZFNs) or transcription activator-like effector nucleases (TALENs), have enabled the study of several disease causing mutations [190,191,192]. Many PSC-lines have been generated by using this technology for both disease modeling and even clinical applications [193,194,195,196]. Vector-mediated delivery of sequence-specific nucleases along with a homologous DNA template to patient-derived iPSCs leads to the excision of targeted locus and, by virtue of cellular homology directed repair (HDR) system, can be corrected by the homologous template with the desired genetic modification. A prominent example is the combination of ZFNs and piggyBac technology which could achieve a biallelic correction of a disease-causing mutation in human iPSCs [197]. In a recent study by Karakikis et al., they reported the use of TALENs to correct gene mutations in patients with hereditary heart failure [198]. These patients harbor an amino acid deletion mutation (R14del) in the coding region of the phospholamban (PLN) gene, which is an important regulator of cardiac calcium cycling in the sarcoplasmic reticulum (SR). They display a phenotype of dilated cardiomyopathy, hypertrophy, episodic ventricular arrhythmia, and overt HF by middle age [199,200]. Skin-derived iPSCs from these patients were isolated, edited, and CM-differentiated, where further analyses showed reversal of the disease’s phenotype. Nevertheless, engineering of sequence-specific ZFNs or TALENs, as well as achieving their robust delivery for this purpose, can be laborious and technically challenging, let alone high in cost [192,194,201]. 

In recent years, CRISPR/Cas9 has emerged as the new horsepower of genome-editing technology, overshadowing ZFNs and TALENs [202]. The system, first described in prokaryotes as part of their adaptive immune system, relies on an RNA-guided endonuclease (Cas9) that localizes to complementary DNA sequences, where it creates double-strand break amenable for correction by the cell’s endogenous HR. Provided that a homologous sequence is available, desired gene modifications can be introduced [202]. Indeed, CRISPR/Cas9 has been zealously received by cell biologists as an attractive tool for SC research [203]. In cardiovascular biology, CRISPR/Cas9 was successfully applied to patient-derived iPSC to target disease-causing mutations of CVDs [204,205,206]. A recent study demonstrated the utility of CRISPR/Cas9 in phenotypic characterization of iPSC-CMs from patients with arrhythmogenic right ventricular dysplasia/cardiomyopathy (ARVD/C) [207]. In this patient cohort, mutations in the *SCN5A* encoding the Na_v_1.5 sodium channel protein led to the phenotype, which could be reversed in this study upon editing with CRISPR/Cas9.

A study published earlier this year by Seeger and colleagues made use of genome-editing techniques to create isogenic iPSC lines from patients with heterozygous mutations in the myosin-binding protein C3 (*MYBPC3*), which is deemed as the underlying cause of hypertrophic cardiomyopathy (HCM) [208]. Their results refuted previous hypotheses of either MZBPC3 haploinsufficiency or truncated poison peptide as the underlying cause of HCM. However, they were able to provide evidence for chronic activation of the nonsense-mediated decay (NMD) as the initial pathogenic trigger that leads to dysregulated gene expression and aberrant calcium signaling upon MYBPC3 mutations.

The aforementioned examples give a great promise to SC therapy of CVDs. One might also envisage the possibility of autologous cell transplantation of iPSC-derived CMs with rectified mutations to ameliorate or even cure disease conditions. However, great challenges remain as to the validation of these technologies, let alone deciding on a safe and effective clinical setting for PSCs delivery to treat CVDs. The next chapter outlines recent advances in preclinical research on SC-based therapy for CVDs.

## 6. Translational Potential of PSCs in Cardiovascular Regenerative Therapy

Harnessing the multifaceted potential of SCs for effective therapeutic purposes to treat CVDs is the ultimate goal of the above-introduced laborious efforts of scientists over the past decades. Provided that SC-derived CMs are sufficiently propagated, differentiated, and maturated, their application to the diseased myocardium spans a wide spectrum of delivery methods, from intravenous administration to direct myocardial injection. Nevertheless, several factors are to be considered with regard to engraftment of transplanted cells and integration, as well as functional contribution to host myocardium, electromechanical coupling between graft and host CMs, and long-term survival. The aforementioned limitations have long been challenges to preclinical and translational applications of SC therapy in general, and in cardiac regenerative therapy in particular. The following summarizes advances made in the realm of preclinical and translational research with PSCs over the past decades, in light of examples from small and large animal models and up until the first clinical initiatives.

### 6.1. Pluripotent Stem Cells in Rodent Models

Earlier studies attempted to engraft human ESC-derived cardiomyocytes in rodent models and reported transient functional improvement in cardiac parameters [209,210]. However, poor engraftment and survival of transplanted cells has been a challenge in these settings. Laflamme et al. utilized pro-survival factor cocktail to limit CM death upon engraftment in infarcted rat heart and reported positive outcomes [108]. To overcome poor engraftment and survival issues, Masumoto and colleagues developed a layered-sheet assembly of three cardiovascular cell populations, namely CMs, endothelial cells, and vascular mural cells, differentiated from mouse ESCs and transplanted into nude-rat model of MI. The transplanted sheets were reported to ameliorate infarct size and improve cardiac function; however, such benefits were shown to be attributed to paracrine-mediated neovascularization and not to actual contribution of transplanted cells [211]. Despite these results, the same group of scientists from Kyoto continued to optimize the stacked-sheet approach, and they recently reported successful long-term survival of engrafted cells through insertion of gelatin hydrogel microspheres between each cardiovascular cell sheet [212]. Analogously, human iPSC-CMs have recently demonstrated favorable therapeutic outcomes when injected in infarcted myocardia of mice. Interestingly, however, the engraftability and survival of those cells depended heavily on the maturation stage [142].

Importantly, electromechanical coupling between the graft and host myocytes is a rather crucial consideration to avoid ventricular arrhythmia. In a Guinea pig model, Shiba et al. reported successful engraftment of human ESC-derived CMs with a 1:1 electrophysiological coupling and improved mechanical function of injured hearts [213].

### 6.2. Pluripotent Stem Cells in Large-Animal Models

#### 6.2.1. Porcine Models

Swine models were featured in the earliest attempts of cell therapy for heart disease. A number of features make the pig an attractive translational model. These include a heart weight-to-body ratio that is equal to a human’s and a similar sinus rate (~90 bpm) [214]. ESC-derived cardiomyocytes have been functionally tested in a swine model of complete atrioventricular block as biologic pacemaker for the treatment of bradycardia [215]. Hereof, Kehat et al. reported survival and functional integration of the transplanted cells, which were able to pace the porcine ventricle with complete heart block [215]. In another porcine model of acute MI, Ye and colleagues used a mixture of cardiovascular cell populations—this time from human iPSCs origin—loaded on a three-dimensional fibrin patch containing IGF-1 (insulin-like growth factor 1) and reported functional integration and significant improvements of several cardiovascular parameters [216]. More recently, Kawamura et al. took a rather unprecedented approach to enhance survival and engraftment of transplanted human iPSC-CMs by combining cell-sheets with pedicle omentum flap as a source of angiogenic factors and reported enhanced engraftment, survival, and therapeutic outcome in a porcine model of ischemic cardiomyopathy [217,218]. The aforementioned tissue sheet technology from Kyoto was also recently applied to a porcine model of MI, where a heterogeneous mixture of cardiovascular cell populations differentiated from human iPSCs and reported functional restoration of the infarcted hearts and attenuated remodeling [219]. Another study from 2018 by Gao and colleagues reported the application of human iPSC-derived fabricated cardiac muscle patches (hCMPs) composed of CMs, smooth muscle and endothelial cells, reprogramed from cardiac fibroblasts and maturated in dynamic culture conditions [220]. They transplanted these patches in infarcted pig hearts and demonstrated significant improvements upon histological and functional analyses. Altogether, these results highlight the importance of co-administration of nonmyocyte cardiac cells, which provide paracrine and angiogenic support equally important to both host and graft tissue. Furthermore, they highlight the superiority of modern tissue engineered scaffolds over direct application of cells. Finally, these studies corroborate the utility of swine models for translational cardiovascular research.

#### 6.2.2. Non-Human Primate Models

The utility of non-human primates (NHPs) in regenerative medicine has long been appreciated, especially in transplantation medicine [221]. In this regard, certain macaque species (e.g., *Macaca fascicularis* or Mauritian Cynomolgus macaque) are a valuable preclinical model to study allogenic transplantation of iPSCs [222]. This is because they exhibit limited diversity in their major histocompatibility complex (MHC) genes, which are distributed only among seven haplotypes and are structurally identical to those in humans [223,224]. Indeed, allogenic transplantation of iPSC-CM among MHC-matched Cynomolgus monkeys was shown in a study by Shiba et al. to be immune-tolerable, and improved cardiac contractile function upon MI [224]. Matching MHC antigens between donors and recipients was shown by others to reduce immunogenicity upon allogenic transplantation of iPSC-CM in the Cynomolgus macaque [225].

NHPs continue to provide unmatched insights to PSC-therapy of CVDs in late-translational studies. In a recently published elegant work by Chong et al., human ESC-CMs were utilized in the pigtail macaque (*Macaca nemestrina*) as a model of ischemia-reperfusion injury [226]. They reported significant re-muscularization of the infarcted areas, structural and functional integration of the grafted cells via establishment of adherent junctions, and electromechanical coupling, typified by synchronous calcium transients. Such promising results were slightly relegated by the presence of arrhythmia in the grafted animals—albeit nonfatal.

## 7. Pluripotent Stem Cells in First Human Trials

The aforementioned successes in late-translational studies with large animal models, as well as the advances made in tissue engineering and grafting techniques, paved the way to the first clinical application of PSC therapy in cardiac settings, which was recently reported in a case study by Menasche et al., using ESC-derived cardiac progenitors [227]. They used the ESC I6 line, which was enriched in vitro by culturing on clinical-grade irradiated human foreskin fibroblasts as feeder cells. Cardiac commitment was then achieved by bone morphogenic protein-2 (BMP-2) and a specific tyrosine kinase inhibitor of the fibroblast growth factor receptor (FGFR), and then confirmed by the expression of the cardiac specific transcription factor Isl-1, as well as the stage-specific embryonic antigen-1 (SSEA-1), which was used for cell purification by immunomagnetic sorting. The cells were embedded in a fibrin scaffold patch and surgically implanted in the infarcted area of a 68-year-old woman patient with severe heart failure. The three-month follow-up showed functional integration of the patch, evident by electrocardiography, and overall symptomatic improvement marked by enhanced left-ventricular ejection fraction (LVEF), with no complications of arrhythmia, tumors, or immunosuppression-related adverse events. The results were encouraging, and the Parisian group conducted a larger-scale study, wherein six patients received cellularized patches of ESC-derived committed cardiac progenitors [228]. Their one-year follow-up demonstrated safety and tolerability of the grafted cells, with no detected tumors. Moreover, they reported modest symptomatic improvements, as well as in different cardiac parameters.

As for iPSCs, a group of scientists from Osaka have reported their granted permission to pursue with their clinical application last year and the results are yet to be reported [229].

## 8. Conclusions and Remarks

Stem cells are a novel source of cells which might be used as a screening tool for pharmaceutical developments. Here, single cells on iPSC status, as well as differentiated cardiomyocyte progenitors, might be used. In addition, engineered heart tissue displays a second model situation for screening of novel therapeutic options, before applying in animal experiments or clinical trials. Since these systems are based on human sources of cells, testing in these model situations might enhance safety and side effect prediction in novel approaches of cardiovascular therapies. Utilization of stem cells in patients suffering from cardiovascular disease is a second interesting field with great potential. The allogenic transplantation of stem cells requires only a modest immunosuppression and might improve cardiac function and thereby survival, as well as quality of life, in patients suffering from cardiac conditions, e.g., heart failure. However, the clinical potentials, as well as the potential side effects, need to be investigated in clinical trials before establishing stem-cell-based therapy as a standard of care in cardiovascular patients. In summary, stem cells, especially induced pluripotent stem cells, have wide therapeutic potential, but need to be characterized and investigated in more detail in preclinical, as well as clinical, trials to understand in more detail their potentials and risks.

## Figures and Tables

**Table 1:** Major structural, electrophysiological and metabolic differences between human PSC-derived CMs and maturated/adulte CMs.

PSC-Derived CM	Mature/Adult CM
Smaller in size, roundish in shape	Larger in size, elongated in shape
Disorganized sarcomeres	Organized sarcomeres
Slow/skeletal troponin I (TnIs)	Adult cardiac troponin I (TnIc)
Titin N2BA isoform	Titin N2B isoform
Higher αMHC:βMHC	Lower αMHC:βMHC
Poor expression of ion-transport components genes (e.g., KCNJ2, RYR2)	High expression of ion-transport components genes
Less efficient Calcium handling	Improved Calcium handling
Less negative resting membrane potential	More negative resting membrane potential
No or few T-tubules	Abundant T-tubules
Few, underdeveloped mitochondria Glucose as major energy source	Dense, well-distributed and developed mitochondria Fatty acids as major energy source

## Abbreviations

CM	cardiomyocytes
CPC	cardiac progenitor cells
CVD	cardiovascular disease
ESC	embryonic stem cells
iPSC	induced pluripotent stem cells
LVEF	left ventricular ejection fraction
MSC	mesenchymal stem cells
PSC	pluripotent stem cells
SM	skeletal myoblast

## References

[1] WHO Cardiovascular Diseases (CVDs). http://www.who.int/en/news-room/fact-sheets/detail/cardiovascular-diseases-.

[2] Benjamin E.J., Blaha M.J., Chiuve S.E., Cushman M., Das S.R., Deo R., de Ferranti S.D., Floyd J., Fornage M., Gillespie C. (2017). Heart Disease and Stroke Statistics-2017 Update: A Report from the American Heart Association. Circulation.

[3] Samak M., Fatullayev J., Sabashnikov A., Zeriouh M., Schmack B., Ruhparwar A., Karck M., Popov A.F., Dohmen P.M., Weymann A. (2016). Total Arterial Revascularization: Bypassing Antiquated Notions to Better Alternatives for Coronary Artery Disease. Med. Sci. Monit. Basic Res..

[4] Fatullayev J., Samak M., Sabashnikov A., Zeriouh M., Rahmanian P.B., Choi Y.H., Schmack B., Kallenbach K., Ruhparwar A., Eghbalzadeh K. (2015). Continuous-Flow Left Ventricular Assist Device Thrombosis: A Danger Foreseen is a Danger Avoided. Med. Sci. Monit. Basic Res..

[5] Roger V.L. (2013). Epidemiology of heart failure. Circ. Res..

[6] Gass A.L., Emaminia A., Lanier G., Aggarwal C., Brown K.A., Raffa M., Kai M., Spielvogel D., Malekan R., Tang G. (2015). Cardiac Transplantation in the New Era. Cardiol. Rev..

[7] Gouadon E., Moore-Morris T., Smit N.W., Chatenoud L., Coronel R., Harding S.E., Jourdon P., Lambert V., Rucker-Martin C., Puceat M. (2016). Concise Review: Pluripotent Stem Cell-Derived Cardiac Cells, A Promising Cell Source for Therapy of Heart Failure: Where Do We Stand?. Stem Cells.

[8] Sampogna G., Guraya S.Y., Forgione A. (2015). Regenerative medicine: Historical roots and potential strategies in modern medicine. J. Microsc. Ultrastruct..

[9] Muller P., Lemcke H., David R. (2018). Stem Cell Therapy in Heart Diseases-Cell Types, Mechanisms and Improvement Strategies. Cell. Physiol. Biochem. Int. J. Exp. Cell. Physiol. Biochem. Pharmacol..

[10] Viola J.L.B., Grad O. (2003). The Emergence of Tissue Engineering as a Research Field.

[11] Tajbakhsh S. (2003). Stem cells to tissue: Molecular, cellular and anatomical heterogeneity in skeletal muscle. Curr. Opin. Genet. Dev..

[12] Al Attar N., Carrion C., Ghostine S., Garcin I., Vilquin J.T., Hagege A.A., Menasche P. (2003). Long-term (1 year) functional and histological results of autologous skeletal muscle cells transplantation in rat. Cardiovasc. Res..

[13] Suzuki K., Murtuza B., Suzuki N., Smolenski R.T., Yacoub M.H. (2001). Intracoronary infusion of skeletal myoblasts improves cardiac function in doxorubicin-induced heart failure. Circulation.

[14] Pouly J., Hagege A.A., Vilquin J.T., Bissery A., Rouche A., Bruneval P., Duboc D., Desnos M., Fiszman M., Fromes Y. (2004). Does the functional efficacy of skeletal myoblast transplantation extend to nonischemic cardiomyopathy?. Circulation.

[15] Gavira J.J., Perez-Ilzarbe M., Abizanda G., Garcia-Rodriguez A., Orbe J., Paramo J.A., Belzunce M., Rabago G., Barba J., Herreros J. (2006). A comparison between percutaneous and surgical transplantation of autologous skeletal myoblasts in a swine model of chronic myocardial infarction. Cardiovasc. Res..

[16] He K.L., Yi G.H., Sherman W., Zhou H., Zhang G.P., Gu A., Kao R., Haimes H.B., Harvey J., Roos E. (2005). Autologous skeletal myoblast transplantation improved hemodynamics and left ventricular function in chronic heart failure dogs. J. Heart Lung Transplant..

[17] Gavira J.J., Nasarre E., Abizanda G., Perez-Ilzarbe M., de Martino-Rodriguez A., Garcia de Jalon J.A., Mazo M., Macias A., Garcia-Bolao I., Pelacho B. (2010). Repeated implantation of skeletal myoblast in a swine model of chronic myocardial infarction. Eur. Heart J..

[18] Menasche P., Hagege A.A., Vilquin J.T., Desnos M., Abergel E., Pouzet B., Bel A., Sarateanu S., Scorsin M., Schwartz K. (2003). Autologous skeletal myoblast transplantation for severe postinfarction left ventricular dysfunction. J. Am. Coll. Cardiol..

[19] Herreros J., Prosper F., Perez A., Gavira J.J., Garcia-Velloso M.J., Barba J., Sanchez P.L., Canizo C., Rabago G., Marti-Climent J.M. (2003). Autologous intramyocardial injection of cultured skeletal muscle-derived stem cells in patients with non-acute myocardial infarction. Eur. Heart J..

[20] Siminiak T., Fiszer D., Jerzykowska O., Grygielska B., Rozwadowska N., Kalmucki P., Kurpisz M. (2005). Percutaneous trans-coronary-venous transplantation of autologous skeletal myoblasts in the treatment of post-infarction myocardial contractility impairment: The POZNAN trial. Eur. Heart J..

[21] Smits P.C., van Geuns R.J., Poldermans D., Bountioukos M., Onderwater E.E., Lee C.H., Maat A.P., Serruys P.W. (2003). Catheter-based intramyocardial injection of autologous skeletal myoblasts as a primary treatment of ischemic heart failure: Clinical experience with six-month follow-up. J. Am. Coll. Cardiol..

[22] Hagege A.A., Marolleau J.P., Vilquin J.T., Alheritiere A., Peyrard S., Duboc D., Abergel E., Messas E., Mousseaux E., Schwartz K. (2006). Skeletal myoblast transplantation in ischemic heart failure: Long-term follow-up of the first phase I cohort of patients. Circulation.

[23] Ince H., Petzsch M., Rehders T.C., Chatterjee T., Nienaber C.A. (2004). Transcatheter transplantation of autologous skeletal myoblasts in postinfarction patients with severe left ventricular dysfunction. J. Endovasc. Ther..

[24] Veltman C.E., Soliman O.I., Geleijnse M.L., Vletter W.B., Smits P.C., ten Cate F.J., Jordaens L.J., Balk A.H., Serruys P.W., Boersma E. (2008). Four-year follow-up of treatment with intramyocardial skeletal myoblasts injection in patients with ischaemic cardiomyopathy. Eur. Heart J..

[25] Reinecke H., Poppa V., Murry C.E. (2002). Skeletal muscle stem cells do not transdifferentiate into cardiomyocytes after cardiac grafting. J. Mol. Cell. Cardiol..

[26] Abraham M.R., Henrikson C.A., Tung L., Chang M.G., Aon M., Xue T., Li R.A., B O.R., Marban E. (2005). Antiarrhythmic engineering of skeletal myoblasts for cardiac transplantation. Circ. Res..

[27] Brickwedel J., Gulbins H., Reichenspurner H. (2014). Long-term follow-up after autologous skeletal myoblast transplantation in ischaemic heart disease. Interact. Cardiovasc. Thorac. Surg..

[28] Dib N., Dinsmore J., Lababidi Z., White B., Moravec S., Campbell A., Rosenbaum A., Seyedmadani K., Jaber W.A., Rizenhour C.S. (2009). One-year follow-up of feasibility and safety of the first U.S., randomized, controlled study using 3-dimensional guided catheter-based delivery of autologous skeletal myoblasts for ischemic cardiomyopathy (CAuSMIC study). JACC Cardiovasc. Interv..

[29] Duckers H.J., Houtgraaf J., Hehrlein C., Schofer J., Waltenberger J., Gershlick A., Bartunek J., Nienaber C., Macaya C., Peters N. (2011). Final results of a phase IIa, randomised, open-label trial to evaluate the percutaneous intramyocardial transplantation of autologous skeletal myoblasts in congestive heart failure patients: The SEISMIC trial. Eurointerv. J. Eur. Collab. Work. Group Interv. Cardiol. Eur. Soc. Cardiol..

[30] Menasche P., Alfieri O., Janssens S., McKenna W., Reichenspurner H., Trinquart L., Vilquin J.T., Marolleau J.P., Seymour B., Larghero J. (2008). The Myoblast Autologous Grafting in Ischemic Cardiomyopathy (MAGIC) trial: First randomized placebo-controlled study of myoblast transplantation. Circulation.

[31] Povsic T.J., O’Connor C.M., Henry T., Taussig A., Kereiakes D.J., Fortuin F.D., Niederman A., Schatz R., Spencer R., Owens D. (2011). A double-blind, randomized, controlled, multicenter study to assess the safety and cardiovascular effects of skeletal myoblast implantation by catheter delivery in patients with chronic heart failure after myocardial infarction. Am. Heart J..

[32] Ferrari G., Cusella-De Angelis G., Coletta M., Paolucci E., Stornaiuolo A., Cossu G., Mavilio F. (1998). Muscle regeneration by bone marrow-derived myogenic progenitors. Science.

[33] Orlic D., Kajstura J., Chimenti S., Jakoniuk I., Anderson S.M., Li B., Pickel J., McKay R., Nadal-Ginard B., Bodine D.M. (2001). Bone marrow cells regenerate infarcted myocardium. Nature.

[34] Strauer B.E., Brehm M., Zeus T., Gattermann N., Hernandez A., Sorg R.V., Kogler G., Wernet P. (2001). Intracoronary, human autologous stem cell transplantation for myocardial regeneration following myocardial infarction. Deutsche Medizinische Wochenschrift 1946.

[35] Wen Y., Chen B., Wang C., Ma X., Gao Q. (2012). Bone marrow-derived mononuclear cell therapy for patients with ischemic heart disease and ischemic heart failure. Expert Opin. Biol. Ther..

[36] De Jong R., Houtgraaf J.H., Samiei S., Boersma E., Duckers H.J. (2014). Intracoronary stem cell infusion after acute myocardial infarction: A meta-analysis and update on clinical trials. Circ. Cardiovasc. Interv..

[37] Malliaras K., Marban E. (2011). Cardiac cell therapy: Where we’ve been, where we are, and where we should be headed. Br. Med. Bull..

[38] Miao C., Lei M., Hu W., Han S., Wang Q. (2017). A brief review: The therapeutic potential of bone marrow mesenchymal stem cells in myocardial infarction. Stem Cell Res. Ther..

[39] Erbs S., Linke A., Schachinger V., Assmus B., Thiele H., Diederich K.W., Hoffmann C., Dimmeler S., Tonn T., Hambrecht R. (2007). Restoration of microvascular function in the infarct-related artery by intracoronary transplantation of bone marrow progenitor cells in patients with acute myocardial infarction: The Doppler Substudy of the Reinfusion of Enriched Progenitor Cells and Infarct Remodeling in Acute Myocardial Infarction (REPAIR-AMI) trial. Circulation.

[40] Asahara T., Kawamoto A., Masuda H. (2011). Concise review: Circulating endothelial progenitor cells for vascular medicine. Stem Cells.

[41] Steinhoff G., Nesteruk J., Wolfien M., Kundt G., Borgermann J., David R., Garbade J., Grosse J., Haverich A., Hennig H. (2017). Cardiac Function Improvement and Bone Marrow Response−: Outcome Analysis of the Randomized PERFECT Phase III Clinical Trial of Intramyocardial CD133(+) Application After Myocardial Infarction. EBioMedicine.

[42] Karantalis V., Schulman I.H., Balkan W., Hare J.M. (2015). Allogeneic cell therapy: A new paradigm in therapeutics. Circ. Res..

[43] Dimmeler S., Zeiher A.M. (2009). Cell therapy of acute myocardial infarction: Open questions. Cardiology.

[44] Kanelidis A.J., Premer C., Lopez J., Balkan W., Hare J.M. (2017). Route of Delivery Modulates the Efficacy of Mesenchymal Stem Cell Therapy for Myocardial Infarction: A Meta-Analysis of Preclinical Studies and Clinical Trials. Circ. Res..

[45] Chen S.L., Fang W.W., Ye F., Liu Y.H., Qian J., Shan S.J., Zhang J.J., Chunhua R.Z., Liao L.M., Lin S. (2004). Effect on left ventricular function of intracoronary transplantation of autologous bone marrow mesenchymal stem cell in patients with acute myocardial infarction. Am. J. Cardiol..

[46] Lee J.W., Lee S.H., Youn Y.J., Ahn M.S., Kim J.Y., Yoo B.S., Yoon J., Kwon W., Hong I.S., Lee K. (2014). A randomized, open-label, multicenter trial for the safety and efficacy of adult mesenchymal stem cells after acute myocardial infarction. J. Korean Med Sci..

[47] Chullikana A., Majumdar A.S., Gottipamula S., Krishnamurthy S., Kumar A.S., Prakash V.S., Gupta P.K. (2015). Randomized, double-blind, phase I/II study of intravenous allogeneic mesenchymal stromal cells in acute myocardial infarction. Cytotherapy.

[48] Gao L.R., Pei X.T., Ding Q.A., Chen Y., Zhang N.K., Chen H.Y., Wang Z.G., Wang Y.F., Zhu Z.M., Li T.C. (2013). A critical challenge: Dosage-related efficacy and acute complication intracoronary injection of autologous bone marrow mesenchymal stem cells in acute myocardial infarction. Int. J. Cardiol..

[49] Hare J.M., Fishman J.E., Gerstenblith G., DiFede Velazquez D.L., Zambrano J.P., Suncion V.Y., Tracy M., Ghersin E., Johnston P.V., Brinker J.A. (2012). Comparison of allogeneic vs. autologous bone marrow-derived mesenchymal stem cells delivered by transendocardial injection in patients with ischemic cardiomyopathy: The POSEIDON randomized trial. JAMA.

[50] Hare J.M., DiFede D.L., Rieger A.C., Florea V., Landin A.M., El-Khorazaty J., Khan A., Mushtaq M., Lowery M.H., Byrnes J.J. (2017). Randomized Comparison of Allogeneic Versus Autologous Mesenchymal Stem Cells for Nonischemic Dilated Cardiomyopathy: POSEIDON-DCM Trial. J. Am. Coll. Cardiol..

[51] Bergmann O., Jovinge S. (2014). Cardiac regeneration in vivo: Mending the heart from within?. Stem Cell Res..

[52] Bergmann O., Zdunek S., Alkass K., Druid H., Bernard S., Frisen J. (2011). Identification of cardiomyocyte nuclei and assessment of ploidy for the analysis of cell turnover. Exp. Cell Res..

[53] Laflamme M.A., Murry C.E. (2011). Heart regeneration. Nature.

[54] Ang K.L., Shenje L.T., Reuter S., Soonpaa M.H., Rubart M., Field L.J., Galinanes M. (2010). Limitations of conventional approaches to identify myocyte nuclei in histologic sections of the heart. Am. J. Physiol. Cell Physiol..

[55] Bergmann O., Bhardwaj R.D., Bernard S., Zdunek S., Barnabe-Heider F., Walsh S., Zupicich J., Alkass K., Buchholz B.A., Druid H. (2009). Evidence for cardiomyocyte renewal in humans. Science.

[56] Mollova M., Bersell K., Walsh S., Savla J., Das L.T., Park S.Y., Silberstein L.E., Dos Remedios C.G., Graham D., Colan S. (2013). Cardiomyocyte proliferation contributes to heart growth in young humans. Proc. Natl. Acad. Sci. USA.

[57] Naqvi N., Li M., Calvert J.W., Tejada T., Lambert J.P., Wu J., Kesteven S.H., Holman S.R., Matsuda T., Lovelock J.D. (2014). A proliferative burst during preadolescence establishes the final cardiomyocyte number. Cell.

[58] Beltrami A.P., Barlucchi L., Torella D., Baker M., Limana F., Chimenti S., Kasahara H., Rota M., Musso E., Urbanek K. (2003). Adult cardiac stem cells are multipotent and support myocardial regeneration. Cell.

[59] Aguilar-Sanchez C., Michael M., Pennings S. (2018). Cardiac Stem Cells in the Postnatal Heart: Lessons from Development. Stem Cells Int..

[60] Hesse M., Fleischmann B.K., Kotlikoff M.I. (2014). Concise review: The role of C-kit expressing cells in heart repair at the neonatal and adult stage. Stem Cells.

[61] Nigro P., Perrucci G.L., Gowran A., Zanobini M., Capogrossi M.C., Pompilio G. (2015). c-kit(+) cells: The tell-tale heart of cardiac regeneration?. Cell. Mol. Life Sci..

[62] Jesty S.A., Steffey M.A., Lee F.K., Breitbach M., Hesse M., Reining S., Lee J.C., Doran R.M., Nikitin A.Y., Fleischmann B.K. (2012). c-kit+ precursors support postinfarction myogenesis in the neonatal, but not adult, heart. Proc. Natl. Acad. Sci. USA.

[63] Nadal-Ginard B., Ellison G.M., Torella D. (2014). The cardiac stem cell compartment is indispensable for myocardial cell homeostasis, repair and regeneration in the adult. Stem Cell Res..

[64] Gude N.A., Firouzi F., Broughton K.M., Ilves K., Nguyen K.P., Payne C.R., Sacchi V., Monsanto M.M., Casillas A.R., Khalafalla F.G. (2018). Cardiac c-Kit Biology Revealed by Inducible Transgenesis. Circ. Res..

[65] Hodgkinson C.P., Gomez J.A., Baksh S.S., Payne A., Schmeckpeper J., Pratt R.E., Dzau V.J. (2018). Insights from molecular signature of in vivo cardiac c-Kit(+) cells following cardiac injury and beta-catenin inhibition. J. Mol. Cell. Cardiol..

[66] Li Y., He L., Huang X., Bhaloo S.I., Zhao H., Zhang S., Pu W., Tian X., Li Y., Liu Q. (2018). Genetic Lineage Tracing of Nonmyocyte Population by Dual Recombinases. Circulation.

[67] Li Y., Lv Z., He L., Huang X., Zhang S., Zhao H., Pu W., Li Y., Yu W., Zhang L. (2019). Genetic Tracing Identifies Early Segregation of the Cardiomyocyte and Nonmyocyte Lineages. Circ. Res..

[68] Elhelaly W.M., Cardoso A.C., Pereira A.H.M., Elnawasany A., Ebrahimi S., Nakada Y., Sadek H.A. (2019). C-Kit Cells Do Not Significantly Contribute to Cardiomyogenesis During Neonatal Heart Regeneration. Circulation.

[69] Tzahor E., Poss K.D. (2017). Cardiac regeneration strategies: Staying young at heart. Science.

[70] Van Berlo J.H., Kanisicak O., Maillet M., Vagnozzi R.J., Karch J., Lin S.C., Middleton R.C., Marban E., Molkentin J.D. (2014). c-kit+ cells minimally contribute cardiomyocytes to the heart. Nature.

[71] Sussman M.A., Murry C.E. (2008). Bones of contention: Marrow-derived cells in myocardial regeneration. J. Mol. Cell. Cardiol..

[72] Evans M.J., Kaufman M.H. (1981). Establishment in culture of pluripotential cells from mouse embryos. Nature.

[73] Thomson J.A., Itskovitz-Eldor J., Shapiro S.S., Waknitz M.A., Swiergiel J.J., Marshall V.S., Jones J.M. (1998). Embryonic stem cell lines derived from human blastocysts. Science.

[74] De Trizio E., Brennan C.S. (2004). The business of human embryonic stem cell research and an international analysis of relevant laws. J. Biolaw Bus..

[75] Jain K.K. (2005). Ethical and regulatory aspects of embryonic stem cell research. Expert Opin. Biol. Ther..

[76] Murry C.E., Keller G. (2008). Differentiation of embryonic stem cells to clinically relevant populations: Lessons from embryonic development. Cell.

[77] Davis R.L., Weintraub H., Lassar A.B. (1987). Expression of a single transfected cDNA converts fibroblasts to myoblasts. Cell.

[78] Takahashi K., Yamanaka S. (2006). Induction of pluripotent stem cells from mouse embryonic and adult fibroblast cultures by defined factors. Cell.

[79] Okita K., Ichisaka T., Yamanaka S. (2007). Generation of germline-competent induced pluripotent stem cells. Nature.

[80] Masip M., Veiga A., Izpisua Belmonte J.C., Simon C. (2010). Reprogramming with defined factors: From induced pluripotency to induced transdifferentiation. Mol. Hum. Reprod..

[81] Zhao Y., Yin X., Qin H., Zhu F., Liu H., Yang W., Zhang Q., Xiang C., Hou P., Song Z. (2008). Two supporting factors greatly improve the efficiency of human iPSC generation. Cell Stem Cell.

[82] Han J., Yuan P., Yang H., Zhang J., Soh B.S., Li P., Lim S.L., Cao S., Tay J., Orlov Y.L. (2010). Tbx3 improves the germ-line competency of induced pluripotent stem cells. Nature.

[83] Takahashi K., Tanabe K., Ohnuki M., Narita M., Ichisaka T., Tomoda K., Yamanaka S. (2007). Induction of pluripotent stem cells from adult human fibroblasts by defined factors. Cell.

[84] Yu J., Vodyanik M.A., Smuga-Otto K., Antosiewicz-Bourget J., Frane J.L., Tian S., Nie J., Jonsdottir G.A., Ruotti V., Stewart R. (2007). Induced pluripotent stem cell lines derived from human somatic cells. Science.

[85] Wernig M., Meissner A., Foreman R., Brambrink T., Ku M., Hochedlinger K., Bernstein B.E., Jaenisch R. (2007). In vitro reprogramming of fibroblasts into a pluripotent ES-cell-like state. Nature.

[86] Maherali N., Sridharan R., Xie W., Utikal J., Eminli S., Arnold K., Stadtfeld M., Yachechko R., Tchieu J., Jaenisch R. (2007). Directly reprogrammed fibroblasts show global epigenetic remodeling and widespread tissue contribution. Cell Stem Cell.

[87] Chin M.H., Mason M.J., Xie W., Volinia S., Singer M., Peterson C., Ambartsumyan G., Aimiuwu O., Richter L., Zhang J. (2009). Induced pluripotent stem cells and embryonic stem cells are distinguished by gene expression signatures. Cell Stem Cell.

[88] Doi A., Park I.H., Wen B., Murakami P., Aryee M.J., Irizarry R., Herb B., Ladd-Acosta C., Rho J., Loewer S. (2009). Differential methylation of tissue- and cancer-specific CpG island shores distinguishes human induced pluripotent stem cells, embryonic stem cells and fibroblasts. Nat. Genet..

[89] Marchetto M.C., Yeo G.W., Kainohana O., Marsala M., Gage F.H., Muotri A.R. (2009). Transcriptional signature and memory retention of human-induced pluripotent stem cells. PLoS ONE.

[90] Kim K., Doi A., Wen B., Ng K., Zhao R., Cahan P., Kim J., Aryee M.J., Ji H., Ehrlich L.I. (2010). Epigenetic memory in induced pluripotent stem cells. Nature.

[91] Hewitt K.J., Garlick J.A. (2013). Cellular reprogramming to reset epigenetic signatures. Mol. Asp. Med..

[92] Gherghiceanu M., Barad L., Novak A., Reiter I., Itskovitz-Eldor J., Binah O., Popescu L.M. (2011). Cardiomyocytes derived from human embryonic and induced pluripotent stem cells: Comparative ultrastructure. J. Cell. Mol. Med..

[93] Gupta M.K., Illich D.J., Gaarz A., Matzkies M., Nguemo F., Pfannkuche K., Liang H., Classen S., Reppel M., Schultze J.L. (2010). Global transcriptional profiles of beating clusters derived from human induced pluripotent stem cells and embryonic stem cells are highly similar. BMC Dev. Biol..

[94] Volarevic V., Markovic B.S., Gazdic M., Volarevic A., Jovicic N., Arsenijevic N., Armstrong L., Djonov V., Lako M., Stojkovic M. (2018). Ethical and Safety Issues of Stem Cell-Based Therapy. Int. J. Med. Sci..

[95] Kiskinis E., Eggan K. (2010). Progress toward the clinical application of patient-specific pluripotent stem cells. J. Clin. Investig..

[96] Kurtz A., Stacey G., Kidane L., Seriola A., Stachelscheid H., Veiga A. (2014). Regulatory insight into the European human pluripotent stem cell registry. Stem Cells Dev..

[97] Lee J.Y., Lee D.Y., Choi Y.S., Lee K.J., Kim Y.O. (2011). Registration of human embryonic stem cell lines: Korea, 2010. Osong Public Health Res. Perspect..

[98] Obama B. (2009). Executive Order 13505 of March 9, 2009: Removing Barriers to Responsible Scientific Research Involving Human Stem Cells. Government Publishing Office. https://www.gpo.gov/fdsys/pkg/FR-2009-03-11/pdf/E9-5441.pdf.

[99] Sanganalmath S.K., Bolli R. (2013). Cell therapy for heart failure: A comprehensive overview of experimental and clinical studies, current challenges, and future directions. Circ. Res..

[100] Olson E.N. (2006). Gene regulatory networks in the evolution and development of the heart. Science.

[101] Costello I., Pimeisl I.M., Drager S., Bikoff E.K., Robertson E.J., Arnold S.J. (2011). The T-box transcription factor Eomesodermin acts upstream of Mesp1 to specify cardiac mesoderm during mouse gastrulation. Nat. Cell Biol..

[102] David R., Jarsch V.B., Schwarz F., Nathan P., Gegg M., Lickert H., Franz W.M. (2011). Induction of MesP1 by Brachyury(T) generates the common multipotent cardiovascular stem cell. Cardiovasc. Res..

[103] Wu S.M., Chien K.R., Mummery C. (2008). Origins and fates of cardiovascular progenitor cells. Cell.

[104] Sylva M., van den Hoff M.J., Moorman A.F. (2014). Development of the human heart. Am. J. Med. Genet. Part A.

[105] De Bakker B.S., de Jong K.H., Hagoort J., de Bree K., Besselink C.T., de Kanter F.E., Veldhuis T., Bais B., Schildmeijer R., Ruijter J.M. (2016). An interactive three-dimensional digital atlas and quantitative database of human development. Science.

[106] Gadue P., Huber T.L., Nostro M.C., Kattman S., Keller G.M. (2005). Germ layer induction from embryonic stem cells. Exp. Hematol..

[107] Moretti A., Laugwitz K.L., Dorn T., Sinnecker D., Mummery C. (2013). Pluripotent stem cell models of human heart disease. Cold Spring Harb. Perspect. Med..

[108] Laflamme M.A., Chen K.Y., Naumova A.V., Muskheli V., Fugate J.A., Dupras S.K., Reinecke H., Xu C., Hassanipour M., Police S. (2007). Cardiomyocytes derived from human embryonic stem cells in pro-survival factors enhance function of infarcted rat hearts. Nat. Biotechnol..

[109] Ren Y., Lee M.Y., Schliffke S., Paavola J., Amos P.J., Ge X., Ye M., Zhu S., Senyei G., Lum L. (2011). Small molecule Wnt inhibitors enhance the efficiency of BMP-4-directed cardiac differentiation of human pluripotent stem cells. J. Mol. Cell. Cardiol..

[110] Zeineddine D., Papadimou E., Mery A., Menard C., Puceat M. (2005). Cardiac commitment of embryonic stem cells for myocardial repair. Methods Mol. Med..

[111] Kehat I., Kenyagin-Karsenti D., Snir M., Segev H., Amit M., Gepstein A., Livne E., Binah O., Itskovitz-Eldor J., Gepstein L. (2001). Human embryonic stem cells can differentiate into myocytes with structural and functional properties of cardiomyocytes. J. Clin. Investig..

[112] Mauritz C., Schwanke K., Reppel M., Neef S., Katsirntaki K., Maier L.S., Nguemo F., Menke S., Haustein M., Hescheler J. (2008). Generation of functional murine cardiac myocytes from induced pluripotent stem cells. Circulation.

[113] Narazaki G., Uosaki H., Teranishi M., Okita K., Kim B., Matsuoka S., Yamanaka S., Yamashita J.K. (2008). Directed and systematic differentiation of cardiovascular cells from mouse induced pluripotent stem cells. Circulation.

[114] Mummery C., Ward-van Oostwaard D., Doevendans P., Spijker R., van den Brink S., Hassink R., van der Heyden M., Opthof T., Pera M., de la Riviere A.B. (2003). Differentiation of human embryonic stem cells to cardiomyocytes: Role of coculture with visceral endoderm-like cells. Circulation.

[115] Burridge P.W., Matsa E., Shukla P., Lin Z.C., Churko J.M., Ebert A.D., Lan F., Diecke S., Huber B., Mordwinkin N.M. (2014). Chemically defined generation of human cardiomyocytes. Nat. Methods.

[116] Lian X., Zhang J., Azarin S.M., Zhu K., Hazeltine L.B., Bao X., Hsiao C., Kamp T.J., Palecek S.P. (2013). Directed cardiomyocyte differentiation from human pluripotent stem cells by modulating Wnt/beta-catenin signaling under fully defined conditions. Nat. Protoc..

[117] Lian X., Bao X., Zilberter M., Westman M., Fisahn A., Hsiao C., Hazeltine L.B., Dunn K.K., Kamp T.J., Palecek S.P. (2015). Chemically defined, albumin-free human cardiomyocyte generation. Nat. Methods.

[118] Yang L., Soonpaa M.H., Adler E.D., Roepke T.K., Kattman S.J., Kennedy M., Henckaerts E., Bonham K., Abbott G.W., Linden R.M. (2008). Human cardiovascular progenitor cells develop from a KDR+ embryonic-stem-cell-derived population. Nature.

[119] Moretti A., Caron L., Nakano A., Lam J.T., Bernshausen A., Chen Y., Qyang Y., Bu L., Sasaki M., Martin-Puig S. (2006). Multipotent embryonic isl1+ progenitor cells lead to cardiac, smooth muscle, and endothelial cell diversification. Cell.

[120] Elliott D.A., Braam S.R., Koutsis K., Ng E.S., Jenny R., Lagerqvist E.L., Biben C., Hatzistavrou T., Hirst C.E., Yu Q.C. (2011). NKX2-5(eGFP/w) hESCs for isolation of human cardiac progenitors and cardiomyocytes. Nat. Methods.

[121] Dubois N.C., Craft A.M., Sharma P., Elliott D.A., Stanley E.G., Elefanty A.G., Gramolini A., Keller G. (2011). SIRPA is a specific cell-surface marker for isolating cardiomyocytes derived from human pluripotent stem cells. Nat. Biotechnol..

[122] Uosaki H., Fukushima H., Takeuchi A., Matsuoka S., Nakatsuji N., Yamanaka S., Yamashita J.K. (2011). Efficient and scalable purification of cardiomyocytes from human embryonic and induced pluripotent stem cells by VCAM1 surface expression. PLoS ONE.

[123] Gallo P., Grimaldi S., Latronico M.V., Bonci D., Pagliuca A., Gallo P., Ausoni S., Peschle C., Condorelli G. (2008). A lentiviral vector with a short troponin-I promoter for tracking cardiomyocyte differentiation of human embryonic stem cells. Gene Ther..

[124] Kita-Matsuo H., Barcova M., Prigozhina N., Salomonis N., Wei K., Jacot J.G., Nelson B., Spiering S., Haverslag R., Kim C. (2009). Lentiviral vectors and protocols for creation of stable hESC lines for fluorescent tracking and drug resistance selection of cardiomyocytes. PLoS ONE.

[125] Tohyama S., Hattori F., Sano M., Hishiki T., Nagahata Y., Matsuura T., Hashimoto H., Suzuki T., Yamashita H., Satoh Y. (2013). Distinct metabolic flow enables large-scale purification of mouse and human pluripotent stem cell-derived cardiomyocytes. Cell Stem Cell.

[126] Zhao Y., Ransom J.F., Li A., Vedantham V., von Drehle M., Muth A.N., Tsuchihashi T., McManus M.T., Schwartz R.J., Srivastava D. (2007). Dysregulation of cardiogenesis, cardiac conduction, and cell cycle in mice lacking miRNA-1-2. Cell.

[127] Fu J.D., Rushing S.N., Lieu D.K., Chan C.W., Kong C.W., Geng L., Wilson K.D., Chiamvimonvat N., Boheler K.R., Wu J.C. (2011). Distinct roles of microRNA-1 and -499 in ventricular specification and functional maturation of human embryonic stem cell-derived cardiomyocytes. PLoS ONE.

[128] Hodgkinson C.P., Kang M.H., Dal-Pra S., Mirotsou M., Dzau V.J. (2015). MicroRNAs and Cardiac Regeneration. Circ. Res..

[129] Yang X., Pabon L., Murry C.E. (2014). Engineering adolescence: Maturation of human pluripotent stem cell-derived cardiomyocytes. Circ. Res..

[130] Friedman C.E., Nguyen Q., Lukowski S.W., Helfer A., Chiu H.S., Miklas J., Levy S., Suo S., Han J.J., Osteil P. (2018). Single-Cell Transcriptomic Analysis of Cardiac Differentiation from Human PSCs Reveals HOPX-Dependent Cardiomyocyte Maturation. Cell Stem Cell.

[131] Churko J.M., Garg P., Treutlein B., Venkatasubramanian M., Wu H., Lee J., Wessells Q.N., Chen S.Y., Chen W.Y., Chetal K. (2018). Defining human cardiac transcription factor hierarchies using integrated single-cell heterogeneity analysis. Nat. Commun..

[132] Lundy S.D., Zhu W.Z., Regnier M., Laflamme M.A. (2013). Structural and functional maturation of cardiomyocytes derived from human pluripotent stem cells. Stem Cells Dev..

[133] Bhavsar P.K., Dhoot G.K., Cumming D.V., Butler-Browne G.S., Yacoub M.H., Barton P.J. (1991). Developmental expression of troponin I isoforms in fetal human heart. FEBS Lett..

[134] Hunkeler N.M., Kullman J., Murphy A.M. (1991). Troponin I isoform expression in human heart. Circ. Res..

[135] Bedada F.B., Chan S.S., Metzger S.K., Zhang L., Zhang J., Garry D.J., Kamp T.J., Kyba M., Metzger J.M. (2014). Acquisition of a quantitative, stoichiometrically conserved ratiometric marker of maturation status in stem cell-derived cardiac myocytes. Stem Cell Rep..

[136] Taegtmeyer H., Sen S., Vela D. (2010). Return to the fetal gene program: A suggested metabolic link to gene expression in the heart. Ann. N. Y. Acad. Sci..

[137] Everett A.W. (1986). Isomyosin expression in human heart in early pre- and post-natal life. J. Mol. Cell. Cardiol..

[138] Weber N., Schwanke K., Greten S., Wendland M., Iorga B., Fischer M., Geers-Knorr C., Hegermann J., Wrede C., Fiedler J. (2016). Stiff matrix induces switch to pure beta-cardiac myosin heavy chain expression in human ESC-derived cardiomyocytes. Basic Res. Cardiol..

[139] Lahmers S., Wu Y., Call D.R., Labeit S., Granzier H. (2004). Developmental control of titin isoform expression and passive stiffness in fetal and neonatal myocardium. Circ. Res..

[140] Xu X.Q., Soo S.Y., Sun W., Zweigerdt R. (2009). Global expression profile of highly enriched cardiomyocytes derived from human embryonic stem cells. Stem Cells.

[141] Blazeski A., Zhu R., Hunter D.W., Weinberg S.H., Boheler K.R., Zambidis E.T., Tung L. (2012). Electrophysiological and contractile function of cardiomyocytes derived from human embryonic stem cells. Prog. Biophys. Mol. Biol..

[142] Funakoshi S., Miki K., Takaki T., Okubo C., Hatani T., Chonabayashi K., Nishikawa M., Takei I., Oishi A., Narita M. (2016). Enhanced engraftment, proliferation, and therapeutic potential in heart using optimized human iPSC-derived cardiomyocytes. Sci. Rep..

[143] Synnergren J., Ameen C., Jansson A., Sartipy P. (2012). Global transcriptional profiling reveals similarities and differences between human stem cell-derived cardiomyocyte clusters and heart tissue. Physiol. Genom..

[144] Lieu D.K., Liu J., Siu C.W., McNerney G.P., Tse H.F., Abu-Khalil A., Huser T., Li R.A. (2009). Absence of transverse tubules contributes to non-uniform Ca(2+) wavefronts in mouse and human embryonic stem cell-derived cardiomyocytes. Stem Cells Dev..

[145] Ramirez-Bergeron D.L., Runge A., Dahl K.D., Fehling H.J., Keller G., Simon M.C. (2004). Hypoxia affects mesoderm and enhances hemangioblast specification during early development. Development.

[146] Lopaschuk G.D., Jaswal J.S. (2010). Energy metabolic phenotype of the cardiomyocyte during development, differentiation, and postnatal maturation. J. Cardiovasc. Pharmacol..

[147] Kim C., Wong J., Wen J., Wang S., Wang C., Spiering S., Kan N.G., Forcales S., Puri P.L., Leone T.C. (2013). Studying arrhythmogenic right ventricular dysplasia with patient-specific iPSCs. Nature.

[148] Mills R.J., Titmarsh D.M., Koenig X., Parker B.L., Ryall J.G., Quaife-Ryan G.A., Voges H.K., Hodson M.P., Ferguson C., Drowley L. (2017). Functional screening in human cardiac organoids reveals a metabolic mechanism for cardiomyocyte cell cycle arrest. Proc. Natl. Acad. Sci. USA.

[149] Hazeltine L.B., Simmons C.S., Salick M.R., Lian X., Badur M.G., Han W., Delgado S.M., Wakatsuki T., Crone W.C., Pruitt B.L. (2012). Effects of substrate mechanics on contractility of cardiomyocytes generated from human pluripotent stem cells. Int. J. Cell Biol..

[150] Kim D.H., Lipke E.A., Kim P., Cheong R., Thompson S., Delannoy M., Suh K.Y., Tung L., Levchenko A. (2010). Nanoscale cues regulate the structure and function of macroscopic cardiac tissue constructs. Proc. Natl. Acad. Sci. USA.

[151] Lee Y.K., Ng K.M., Chan Y.C., Lai W.H., Au K.W., Ho C.Y., Wong L.Y., Lau C.P., Tse H.F., Siu C.W. (2010). Triiodothyronine promotes cardiac differentiation and maturation of embryonic stem cells via the classical genomic pathway. Mol. Endocrinol..

[152] McDevitt T.C., Laflamme M.A., Murry C.E. (2005). Proliferation of cardiomyocytes derived from human embryonic stem cells is mediated via the IGF/PI 3-kinase/Akt signaling pathway. J. Mol. Cell. Cardiol..

[153] Zimmermann W.H., Schneiderbanger K., Schubert P., Didie M., Munzel F., Heubach J.F., Kostin S., Neuhuber W.L., Eschenhagen T. (2002). Tissue engineering of a differentiated cardiac muscle construct. Circ. Res..

[154] Zimmermann W.H., Melnychenko I., Wasmeier G., Didie M., Naito H., Nixdorff U., Hess A., Budinsky L., Brune K., Michaelis B. (2006). Engineered heart tissue grafts improve systolic and diastolic function in infarcted rat hearts. Nat. Med..

[155] Eschenhagen T., Eder A., Vollert I., Hansen A. (2012). Physiological aspects of cardiac tissue engineering. Am. J. Physiol. Heart Circ. Physiol..

[156] Cui H., Miao S., Esworthy T., Zhou X., Lee S.J., Liu C., Yu Z.X., Fisher J.P., Mohiuddin M., Zhang L.G. (2018). 3D bioprinting for cardiovascular regeneration and pharmacology. Adv. Drug Deliv. Rev..

[157] Sheehy S.P., Grosberg A., Qin P., Behm D.J., Ferrier J.P., Eagleson M.A., Nesmith A.P., Krull D., Falls J.G., Campbell P.H. (2017). Toward improved myocardial maturity in an organ-on-chip platform with immature cardiac myocytes. Exp. Biol. Med..

[158] Schwan J., Kwaczala A.T., Ryan T.J., Bartulos O., Ren Y., Sewanan L.R., Morris A.H., Jacoby D.L., Qyang Y., Campbell S.G. (2016). Anisotropic engineered heart tissue made from laser-cut decellularized myocardium. Sci. Rep..

[159] Gonzalez F., Boue S., Izpisua Belmonte J.C. (2011). Methods for making induced pluripotent stem cells: Reprogramming a la carte. Nat. Rev. Genet..

[160] Yoshida Y., Yamanaka S. (2017). Induced Pluripotent Stem Cells 10 Years Later: For Cardiac Applications. Circ. Res..

[161] Bellin M., Mummery C.L. (2016). Inherited heart disease—What can we expect from the second decade of human iPS cell research?. FEBS Lett..

[162] Carvajal-Vergara X., Sevilla A., D’Souza S.L., Ang Y.S., Schaniel C., Lee D.F., Yang L., Kaplan A.D., Adler E.D., Rozov R. (2010). Patient-specific induced pluripotent stem-cell-derived models of LEOPARD syndrome. Nature.

[163] Dudek J., Cheng I.F., Balleininger M., Vaz F.M., Streckfuss-Bomeke K., Hubscher D., Vukotic M., Wanders R.J., Rehling P., Guan K. (2013). Cardiolipin deficiency affects respiratory chain function and organization in an induced pluripotent stem cell model of Barth syndrome. Stem Cell Res..

[164] Fatima A., Xu G., Shao K., Papadopoulos S., Lehmann M., Arnaiz-Cot J.J., Rosa A.O., Nguemo F., Matzkies M., Dittmann S. (2011). In vitro modeling of ryanodine receptor 2 dysfunction using human induced pluripotent stem cells. Cell. Physiol. Biochem. Int. J. Exp. Cell. Physiol. Biochem. Pharmacol..

[165] Hinson J.T., Chopra A., Nafissi N., Polacheck W.J., Benson C.C., Swist S., Gorham J., Yang L., Schafer S., Sheng C.C. (2015). HEART DISEASE. Titin mutations in iPS cells define sarcomere insufficiency as a cause of dilated cardiomyopathy. Science.

[166] Huang H.P., Chen P.H., Hwu W.L., Chuang C.Y., Chien Y.H., Stone L., Chien C.L., Li L.T., Chiang S.C., Chen H.F. (2011). Human Pompe disease-induced pluripotent stem cells for pathogenesis modeling, drug testing and disease marker identification. Hum. Mol. Genet..

[167] Itzhaki I., Maizels L., Huber I., Zwi-Dantsis L., Caspi O., Winterstern A., Feldman O., Gepstein A., Arbel G., Hammerman H. (2011). Modelling the long QT syndrome with induced pluripotent stem cells. Nature.

[168] Kujala K., Paavola J., Lahti A., Larsson K., Pekkanen-Mattila M., Viitasalo M., Lahtinen A.M., Toivonen L., Kontula K., Swan H. (2012). Cell model of catecholaminergic polymorphic ventricular tachycardia reveals early and delayed afterdepolarizations. PLoS ONE.

[169] Lahti A.L., Kujala V.J., Chapman H., Koivisto A.P., Pekkanen-Mattila M., Kerkela E., Hyttinen J., Kontula K., Swan H., Conklin B.R. (2012). Model for long QT syndrome type 2 using human iPS cells demonstrates arrhythmogenic characteristics in cell culture. Dis. Models Mech..

[170] Ma D., Wei H., Lu J., Ho S., Zhang G., Sun X., Oh Y., Tan S.H., Ng M.L., Shim W. (2013). Generation of patient-specific induced pluripotent stem cell-derived cardiomyocytes as a cellular model of arrhythmogenic right ventricular cardiomyopathy. Eur. Heart J..

[171] Ma D., Wei H., Zhao Y., Lu J., Li G., Sahib N.B., Tan T.H., Wong K.Y., Shim W., Wong P. (2013). Modeling type 3 long QT syndrome with cardiomyocytes derived from patient-specific induced pluripotent stem cells. Int. J. Cardiol..

[172] Moretti A., Bellin M., Welling A., Jung C.B., Lam J.T., Bott-Flugel L., Dorn T., Goedel A., Hohnke C., Hofmann F. (2010). Patient-specific induced pluripotent stem-cell models for long-QT syndrome. N. Engl. J. Med..

[173] Yazawa M., Hsueh B., Jia X., Pasca A.M., Bernstein J.A., Hallmayer J., Dolmetsch R.E. (2011). Using induced pluripotent stem cells to investigate cardiac phenotypes in Timothy syndrome. Nature.

[174] Ojala M., Prajapati C., Polonen R.P., Rajala K., Pekkanen-Mattila M., Rasku J., Larsson K., Aalto-Setala K. (2016). Mutation-Specific Phenotypes in hiPSC-Derived Cardiomyocytes Carrying Either Myosin-Binding Protein C Or alpha-Tropomyosin Mutation for Hypertrophic Cardiomyopathy. Stem Cells Int..

[175] Wang G., McCain M.L., Yang L., He A., Pasqualini F.S., Agarwal A., Yuan H., Jiang D., Zhang D., Zangi L. (2014). Modeling the mitochondrial cardiomyopathy of Barth syndrome with induced pluripotent stem cell and heart-on-chip technologies. Nat. Med..

[176] Sun N., Yazawa M., Liu J., Han L., Sanchez-Freire V., Abilez O.J., Navarrete E.G., Hu S., Wang L., Lee A. (2012). Patient-specific induced pluripotent stem cells as a model for familial dilated cardiomyopathy. Sci. Transl. Med..

[177] Sharma A., Marceau C., Hamaguchi R., Burridge P.W., Rajarajan K., Churko J.M., Wu H., Sallam K.I., Matsa E., Sturzu A.C. (2014). Human induced pluripotent stem cell-derived cardiomyocytes as an in vitro model for coxsackievirus B3-induced myocarditis and antiviral drug screening platform. Circ. Res..

[178] Gu M., Shao N.Y., Sa S., Li D., Termglinchan V., Ameen M., Karakikes I., Sosa G., Grubert F., Lee J. (2017). Patient-Specific iPSC-Derived Endothelial Cells Uncover Pathways that Protect against Pulmonary Hypertension in BMPR2 Mutation Carriers. Cell Stem Cell.

[179] Fatima A., Kaifeng S., Dittmann S., Xu G., Gupta M.K., Linke M., Zechner U., Nguemo F., Milting H., Farr M. (2013). The disease-specific phenotype in cardiomyocytes derived from induced pluripotent stem cells of two long QT syndrome type 3 patients. PLoS ONE.

[180] Paci M., Hyttinen J., Aalto-Setala K., Severi S. (2013). Computational models of ventricular- and atrial-like human induced pluripotent stem cell derived cardiomyocytes. Ann. Biomed. Eng..

[181] Paci M., Passini E., Severi S., Hyttinen J., Rodriguez B. (2017). Phenotypic variability in LQT3 human induced pluripotent stem cell-derived cardiomyocytes and their response to antiarrhythmic pharmacologic therapy: An in silico approach. Heart Rhythm.

[182] Paci M., Polonen R.P., Cori D., Penttinen K., Aalto-Setala K., Severi S., Hyttinen J. (2018). Automatic Optimization of an in Silico Model of Human iPSC Derived Cardiomyocytes Recapitulating Calcium Handling Abnormalities. Front. Physiol..

[183] Bellin M., Casini S., Davis R.P., D’Aniello C., Haas J., Ward-van Oostwaard D., Tertoolen L.G., Jung C.B., Elliott D.A., Welling A. (2013). Isogenic human pluripotent stem cell pairs reveal the role of a KCNH2 mutation in long-QT syndrome. EMBO J..

[184] Rampe D., Brown A.M. (2013). A history of the role of the hERG channel in cardiac risk assessment. J. Pharmacol. Toxicol. Methods.

[185] Ahola A., Polonen R.P., Aalto-Setala K., Hyttinen J. (2018). Simultaneous Measurement of Contraction and Calcium Transients in Stem Cell Derived Cardiomyocytes. Ann. Biomed. Eng..

[186] Bjork S., Ojala E.A., Nordstrom T., Ahola A., Liljestrom M., Hyttinen J., Kankuri E., Mervaala E. (2017). Evaluation of Optogenetic Electrophysiology Tools in Human Stem Cell-Derived Cardiomyocytes. Front. Physiol..

[187] Klimas A., Ambrosi C.M., Yu J., Williams J.C., Bien H., Entcheva E. (2016). OptoDyCE as an automated system for high-throughput all-optical dynamic cardiac electrophysiology. Nat. Commun..

[188] Rajamohan D., Kalra S., Duc Hoang M., George V., Staniforth A., Russell H., Yang X., Denning C. (2016). Automated Electrophysiological and Pharmacological Evaluation of Human Pluripotent Stem Cell-Derived Cardiomyocytes. Stem Cells Dev..

[189] Chi K.R. (2013). Revolution dawning in cardiotoxicity testing. Nat. Rev. Drug Discov..

[190] Miller J.C., Tan S., Qiao G., Barlow K.A., Wang J., Xia D.F., Meng X., Paschon D.E., Leung E., Hinkley S.J. (2011). A TALE nuclease architecture for efficient genome editing. Nat. Biotechnol..

[191] Urnov F.D., Rebar E.J., Holmes M.C., Zhang H.S., Gregory P.D. (2010). Genome editing with engineered zinc finger nucleases. Nat. Rev. Genet..

[192] Kim H., Kim J.S. (2014). A guide to genome engineering with programmable nucleases. Nat. Rev. Genet..

[193] Collin J., Lako M. (2011). Concise review: Putting a finger on stem cell biology: Zinc finger nuclease-driven targeted genetic editing in human pluripotent stem cells. Stem Cells.

[194] Zou J., Maeder M.L., Mali P., Pruett-Miller S.M., Thibodeau-Beganny S., Chou B.K., Chen G., Ye Z., Park I.H., Daley G.Q. (2009). Gene targeting of a disease-related gene in human induced pluripotent stem and embryonic stem cells. Cell Stem Cell.

[195] Hockemeyer D., Soldner F., Beard C., Gao Q., Mitalipova M., DeKelver R.C., Katibah G.E., Amora R., Boydston E.A., Zeitler B. (2009). Efficient targeting of expressed and silent genes in human ESCs and iPSCs using zinc-finger nucleases. Nat. Biotechnol..

[196] Hockemeyer D., Wang H., Kiani S., Lai C.S., Gao Q., Cassady J.P., Cost G.J., Zhang L., Santiago Y., Miller J.C. (2011). Genetic engineering of human pluripotent cells using TALE nucleases. Nat. Biotechnol..

[197] Yusa K., Rashid S.T., Strick-Marchand H., Varela I., Liu P.Q., Paschon D.E., Miranda E., Ordonez A., Hannan N.R., Rouhani F.J. (2011). Targeted gene correction of alpha1-antitrypsin deficiency in induced pluripotent stem cells. Nature.

[198] Karakikes I., Stillitano F., Nonnenmacher M., Tzimas C., Sanoudou D., Termglinchan V., Kong C.W., Rushing S., Hansen J., Ceholski D. (2015). Correction of human phospholamban R14del mutation associated with cardiomyopathy using targeted nucleases and combination therapy. Nat. Commun..

[199] Haghighi K., Kolokathis F., Gramolini A.O., Waggoner J.R., Pater L., Lynch R.A., Fan G.C., Tsiapras D., Parekh R.R., Dorn G.W. (2006). A mutation in the human phospholamban gene, deleting arginine 14, results in lethal, hereditary cardiomyopathy. Proc. Natl. Acad. Sci. USA.

[200] Van der Zwaag P.A., van Rijsingen I.A., Asimaki A., Jongbloed J.D., van Veldhuisen D.J., Wiesfeld A.C., Cox M.G., van Lochem L.T., de Boer R.A., Hofstra R.M. (2012). Phospholamban R14del mutation in patients diagnosed with dilated cardiomyopathy or arrhythmogenic right ventricular cardiomyopathy: Evidence supporting the concept of arrhythmogenic cardiomyopathy. Eur. J. Heart Fail..

[201] Ovando-Roche P., Georgiadis A., Smith A.J., Pearson R.A., Ali R.R. (2017). Harnessing the Potential of Human Pluripotent Stem Cells and Gene Editing for the Treatment of Retinal Degeneration. Curr. Stem Cell Rep..

[202] Hsu P.D., Lander E.S., Zhang F. (2014). Development and applications of CRISPR-Cas9 for genome engineering. Cell.

[203] Chaterji S., Ahn E.H., Kim D.H. (2017). CRISPR Genome Engineering for Human Pluripotent Stem Cell Research. Theranostics.

[204] Limpitikul W.B., Dick I.E., Tester D.J., Boczek N.J., Limphong P., Yang W., Choi M.H., Babich J., DiSilvestre D., Kanter R.J. (2017). A Precision Medicine Approach to the Rescue of Function on Malignant Calmodulinopathic Long-QT Syndrome. Circ. Res..

[205] Yamamoto Y., Makiyama T., Harita T., Sasaki K., Wuriyanghai Y., Hayano M., Nishiuchi S., Kohjitani H., Hirose S., Chen J. (2017). Allele-specific ablation rescues electrophysiological abnormalities in a human iPS cell model of long-QT syndrome with a CALM2 mutation. Hum. Mol. Genet..

[206] Brodehl A., Ebbinghaus H., Deutsch M.A., Gummert J., Gartner A., Ratnavadivel S., Milting H. (2019). Human Induced Pluripotent Stem-Cell-Derived Cardiomyocytes as Models for Genetic Cardiomyopathies. Int. J. Mol. Sci..

[207] Te Riele A.S., Agullo-Pascual E., James C.A., Leo-Macias A., Cerrone M., Zhang M., Lin X., Lin B., Sobreira N.L., Amat-Alarcon N. (2017). Multilevel analyses of SCN5A mutations in arrhythmogenic right ventricular dysplasia/cardiomyopathy suggest non-canonical mechanisms for disease pathogenesis. Cardiovasc. Res..

[208] Seeger T., Shrestha R., Lam C.K., Chen C., McKeithan W.L., Lau E., Wnorowski A., McMullen G., Greenhaw M., Lee J. (2019). A Premature Termination Codon Mutation in MYBPC3 Causes Hypertrophic Cardiomyopathy via Chronic Activation of Nonsense-Mediated Decay. Circulation.

[209] Van Laake L.W., Passier R., Monshouwer-Kloots J., Verkleij A.J., Lips D.J., Freund C., den Ouden K., Ward-van Oostwaard D., Korving J., Tertoolen L.G. (2007). Human embryonic stem cell-derived cardiomyocytes survive and mature in the mouse heart and transiently improve function after myocardial infarction. Stem Cell Res..

[210] Caspi O., Huber I., Kehat I., Habib M., Arbel G., Gepstein A., Yankelson L., Aronson D., Beyar R., Gepstein L. (2007). Transplantation of human embryonic stem cell-derived cardiomyocytes improves myocardial performance in infarcted rat hearts. J. Am. Coll. Cardiol..

[211] Masumoto H., Matsuo T., Yamamizu K., Uosaki H., Narazaki G., Katayama S., Marui A., Shimizu T., Ikeda T., Okano T. (2012). Pluripotent stem cell-engineered cell sheets reassembled with defined cardiovascular populations ameliorate reduction in infarct heart function through cardiomyocyte-mediated neovascularization. Stem Cells.

[212] Matsuo T., Masumoto H., Tajima S., Ikuno T., Katayama S., Minakata K., Ikeda T., Yamamizu K., Tabata Y., Sakata R. (2015). Efficient long-term survival of cell grafts after myocardial infarction with thick viable cardiac tissue entirely from pluripotent stem cells. Sci. Rep..

[213] Shiba Y., Fernandes S., Zhu W.Z., Filice D., Muskheli V., Kim J., Palpant N.J., Gantz J., Moyes K.W., Reinecke H. (2012). Human ES-cell-derived cardiomyocytes electrically couple and suppress arrhythmias in injured hearts. Nature.

[214] Lelovas P.P., Kostomitsopoulos N.G., Xanthos T.T. (2014). A comparative anatomic and physiologic overview of the porcine heart. J. Am. Assoc. Lab. Anim. Sci..

[215] Kehat I., Khimovich L., Caspi O., Gepstein A., Shofti R., Arbel G., Huber I., Satin J., Itskovitz-Eldor J., Gepstein L. (2004). Electromechanical integration of cardiomyocytes derived from human embryonic stem cells. Nat. Biotechnol..

[216] Ye L., Chang Y.H., Xiong Q., Zhang P., Zhang L., Somasundaram P., Lepley M., Swingen C., Su L., Wendel J.S. (2014). Cardiac repair in a porcine model of acute myocardial infarction with human induced pluripotent stem cell-derived cardiovascular cells. Cell Stem Cell.

[217] Kawamura M., Miyagawa S., Fukushima S., Saito A., Miki K., Ito E., Sougawa N., Kawamura T., Daimon T., Shimizu T. (2013). Enhanced survival of transplanted human induced pluripotent stem cell-derived cardiomyocytes by the combination of cell sheets with the pedicled omental flap technique in a porcine heart. Circulation.

[218] Kawamura M., Miyagawa S., Fukushima S., Saito A., Miki K., Funakoshi S., Yoshida Y., Yamanaka S., Shimizu T., Okano T. (2017). Enhanced Therapeutic Effects of Human iPS Cell Derived-Cardiomyocyte by Combined Cell-Sheets with Omental Flap Technique in Porcine Ischemic Cardiomyopathy Model. Sci. Rep..

[219] Ishigami M., Masumoto H., Ikuno T., Aoki T., Kawatou M., Minakata K., Ikeda T., Sakata R., Yamashita J.K., Minatoya K. (2018). Human iPS cell-derived cardiac tissue sheets for functional restoration of infarcted porcine hearts. PLoS ONE.

[220] Gao L., Gregorich Z.R., Zhu W., Mattapally S., Oduk Y., Lou X., Kannappan R., Borovjagin A.V., Walcott G.P., Pollard A.E. (2018). Large Cardiac Muscle Patches Engineered from Human Induced-Pluripotent Stem Cell-Derived Cardiac Cells Improve Recovery from Myocardial Infarction in Swine. Circulation.

[221] Bontrop R.E. (2001). Non-human primates: Essential partners in biomedical research. Immunol. Rev..

[222] Ishigaki H., Shiina T., Ogasawara K. (2018). MHC-identical and transgenic cynomolgus macaques for preclinical studies. Inflamm. Regen..

[223] Deleidi M., Hargus G., Hallett P., Osborn T., Isacson O. (2011). Development of histocompatible primate-induced pluripotent stem cells for neural transplantation. Stem Cells.

[224] Shiba Y., Gomibuchi T., Seto T., Wada Y., Ichimura H., Tanaka Y., Ogasawara T., Okada K., Shiba N., Sakamoto K. (2016). Allogeneic transplantation of iPS cell-derived cardiomyocytes regenerates primate hearts. Nature.

[225] Kawamura T., Miyagawa S., Fukushima S., Maeda A., Kashiyama N., Kawamura A., Miki K., Okita K., Yoshida Y., Shiina T. (2016). Cardiomyocytes Derived from MHC-Homozygous Induced Pluripotent Stem Cells Exhibit Reduced Allogeneic Immunogenicity in MHC-Matched Non-human Primates. Stem Cell Rep..

[226] Chong J.J., Yang X., Don C.W., Minami E., Liu Y.W., Weyers J.J., Mahoney W.M., Van Biber B., Cook S.M., Palpant N.J. (2014). Human embryonic-stem-cell-derived cardiomyocytes regenerate non-human primate hearts. Nature.

[227] Menasche P., Vanneaux V., Hagege A., Bel A., Cholley B., Cacciapuoti I., Parouchev A., Benhamouda N., Tachdjian G., Tosca L. (2015). Human embryonic stem cell-derived cardiac progenitors for severe heart failure treatment: First clinical case report. Eur. Heart J..

[228] Menasche P., Vanneaux V., Hagege A., Bel A., Cholley B., Parouchev A., Cacciapuoti I., Al-Daccak R., Benhamouda N., Blons H. (2018). Transplantation of Human Embryonic Stem Cell-Derived Cardiovascular Progenitors for Severe Ischemic Left Ventricular Dysfunction. J. Am. Coll. Cardiol..

[229] Faculty of Medicine, Osaka University iPS Cell-Based Therapy for Heart Disease: Clinical Application iPS Cell-Derived Cardiomyocytes. http://www.med.osaka-u.ac.jp/eng/archives/2777.

